# Supplementary material for: Assessing trends and reasons for unsuccessful implant discontinuation in Burkina Faso and Kenya between 2016 and 2020: a cross-sectional study
Source: BMJ Open. 2023 Jul 18;13(7):e071775. doi: 10.1136/bmjopen-2023-071775 (PMC10357675; doi:10.1136/bmjopen-2023-071775)
Supplement: Supplementary data [file bmjopen-2023-071775supp001.pdf]

Supplemental Table 1. Characteristics of modern method users compared to implant users, in Burkina Faso and Kenya. 2019.

	Burkina Faso			Kenya		
	2020			2019		
	All Other Modern	Implant	*p-value	All Other Modern	Implant	*p-value
**N**	900	778		2463	1575	
**Total % of all women**	14%	12%		26%	17%	
**Age**			0.300			<0.001
15-24	31%	29%		25%	25%	
25-39	55%	50%		55%	61%	
40-49	14%	22%		20%	14%	
**Marital status**			<0.001			0.001
In union	71%	82%		69%	78%	
Sexually active unmarried	29%	18%		31%	22%	
**Parity**			<0.001			<0.001
0	17%	4%		11%	1%	
1 to 2	29%	30%		37%	42%	
3 to 4	27%	25%		31%	34%	
5 or more	27%	41%		21%	23%	
**Residence**			<0.001			<0.001
Urban	37%	22%		36%	27%	
Rural	63%	78%		64%	73%	
**Education**			<0.001			<0.001
No education	46%	64%		2%	4%	
Primary	19%	19%		49%	57%	
Secondary	30%	16%		37%	30%	
Tertiary	4%	1%		13%	9%	
**Wealth tertile**			<0.001			<0.001
Lowest	25%	34%		28%	38%	
Middle	28%	34%		37%	36%	
Highest	47%	32%		35%	26%	
**Received from public facility**			<0.001			<0.001
Yes	74%	91%		61%	88%	
No	26%	9%		39%	12%	
**Fertility preferences**			0.090			<0.001
No more children	18%	25%		44%	48%	
Children in the next 2 years	35%	31%		16%	13%	
Children more than 2 years from now	38%	34%		33%	34%	
Says she can't get pregnant	1%	1%		2%	1%	
Undecided	8%	9%		5%	3%	
Other	1%	0%		0%	0%	

**We use the chi-square test to assess whether the distribution of characteristics differed based on whether a woman reported implant versus other modern method use.*

Supplemental Table 2. Contraceptive prevalence and method mix over time, among women of reproductive age in Burkina Faso and Kenya. 2016-2020

	Burkina Faso				Kenya			
	2017	2018	2019	2020	2016	2017	2018	2019
**N**	3221	3512	3329	6590	5891	5864	5658	9469
**Current contraceptive use**								
Yes	23%	28%	29%	27%	45%	45%	46%	45%
No	77%	72%	71%	73%	55%	55%	54%	55%
**Current modern contraceptive use**								
Yes	22%	26%	27%	25%	44%	44%	45%	43%
No	78%	74%	73%	75%	56%	56%	55%	57%
**N**	735	982	960	1800	2659	2592	2566	4271
**Method mix***								
Sterilization	0%	0%	0%	0%	4%	4%	4%	4%
IUD	3%	4%	4%	4%	5%	2%	4%	4%
Implant	43%	45%	39%	43%	28%	32%	34%	36%
Injectable	30%	25%	26%	24%	42%	41%	39%	33%
Pills	11%	11%	10%	9%	8%	8%	7%	7%
Condoms	8%	8%	13%	12%	6%	5%	5%	7%
Standard Days Method	0%	1%	0%	0%	0%	1%	0%	0%
Rhythm	3%	5%	5%	6%	2%	2%	2%	3%
Withdrawal	0%	0%	0%	1%	1%	1%	1%	1%
Emergency contraception	0%	0%	1%	0%	2%	3%	3%	2%
Other modern method	0%	0%	0%	1%	1%	0%	0%	1%
Other	1%	0%	1%	0%	0%	0%	0%	2%

*Among women reporting use of more than one method, only the most effective method is included in the calculation of the method mix.
