# Supplementary material for: Assessing trends and reasons for unsuccessful implant discontinuation in Burkina Faso and Kenya between 2016 and 2020: a cross-sectional study
Source: BMJ Open. 2023 Jul 18;13(7):e071775. doi: 10.1136/bmjopen-2023-071775 (PMC10357675; doi:10.1136/bmjopen-2023-071775)
Supplement: Author's manuscript [file bmjopen-2023-071775.draft_revisions.pdf]

BMJ Open is committed to open peer review. As part of this commitment we make the peer review history of every article we publish publicly available.

When an article is published we post the peer reviewers' comments and the authors' responses online. We also post the versions of the paper that were used during peer review. These are the versions that the peer review comments apply to.

The versions of the paper that follow are the versions that were submitted during the peer review process. They are not the versions of record or the final published versions. They should not be cited or distributed as the published version of this manuscript.

BMJ Open is an open access journal and the full, final, typeset and author-corrected version of record of the manuscript is available on our site with no access controls, subscription charges or pay-per-view fees (http://bmjopen.bmj.com).

If you have any questions on BMJ Open's open peer review process please email info.bmjopen@bmj.com

BMJ Open

Barriers to successful implant removal in Burkina Faso and Kenya: Understanding trends and reasons for unsuccessful removal between 2016 and 2020

Journal:	*BMJ Open*
Manuscript ID	bmjopen-2023-071775
Article Type:	Original research
Date Submitted by the Author:	11-Jan-2023

Complete List of Authors:	Tumlinson, Katherine; The University of North Carolina at Chapel Hill, Department of Maternal and Child Health<br>Senderowicz, Leigh ; University of Wisconsin-Madison, Department of Gender and Women's Studies<br>Bullington, Brooke; The University of North Carolina at Chapel Hill, Department of Epidemiology<br>Chung, Stephanie; The University of North Carolina at Chapel Hill, Department of Maternal and Child Health<br>Goland, Emilia; The University of North Carolina at Chapel Hill, Department of Maternal and Child Health<br>Zimmerman, Linnea; Johns Hopkins University Bloomberg School of Public Health, Department of Population, Family & Reproductive Health, Johns Hopkins Bloomberg School of Public Health, Baltimore, MD, USA<br>Gichangi, Peter; International Centre for Reproductive Health Kenya; University of Nairobi<br>Thiongo, Mary; International Centre for Reproductive Health Kenya<br>Guiella, Georges; ISSP/University of Ouagadougou)<br>NA, PMA Principal Investigators Group; NA<br>Karp, Celia; Johns Hopkins University Bloomberg School of Public Health

Keywords:	REPRODUCTIVE MEDICINE, PUBLIC HEALTH, EPIDEMIOLOGY, Quality in health care < HEALTH SERVICES ADMINISTRATION & MANAGEMENT

SCHOLARONE™ Manuscripts

I, the Submitting Author has the right to grant and does grant on behalf of all authors of the Work (as defined in the below author licence), an exclusive licence and/or a non-exclusive licence for contributions from authors who are: i) UK Crown employees; ii) where BMJ has agreed a CC-BY licence shall apply, and/or iii) in accordance with the terms applicable for US Federal Government officers or employees acting as part of their official duties; on a worldwide, perpetual, irrevocable, royalty-free basis to BMJ Publishing Group Ltd ("BMJ") its licensees and where the relevant Journal is co-owned by BMJ to the co-owners of the Journal, to publish the Work in this journal and any other BMJ products and to exploit all rights, as set out in our licence.

The Submitting Author accepts and understands that any supply made under these terms is made by BMJ to the Submitting Author unless you are acting as an employee on behalf of your employer or a postgraduate student of an affiliated institution which is paying any applicable article publishing charge ("APC") for Open Access articles. Where the Submitting Author wishes to make the Work available on an Open Access basis (and intends to pay the relevant APC), the terms of reuse of such Open Access shall be governed by a Creative Commons licence – details of these licences and which Creative Commons licence will apply to this Work are set out in our licence referred to above.

Other than as permitted in any relevant BMJ Author's Self Archiving Policies, I confirm this Work has not been accepted for publication elsewhere, is not being considered for publication elsewhere and does not duplicate material already published. I confirm all authors consent to publication of this Work and authorise the granting of this licence.

For peer review only

Title: Barriers to successful implant removal in Burkina Faso and Kenya: Understanding trends and reasons for unsuccessful removal between 2016 and 2020

Authors: Katherine Tumlinson[1,2], Leigh Senderowicz[3], Brooke W. Bullington[2,4], Stephanie Chung[1], Emilia Goland[1], Linnea Zimmerman[5], Peter Gichangi[6], Mary Thiongo[6], Georges Guiella[7], PMA Principal Investigators Group[#], Celia Karp[5]

Affiliations:
[1] Department of Maternal and Child Health, Gillings School of Global Public Health, University of North Carolina at Chapel Hill
[2] Carolina Population Center, University of North Carolina at Chapel Hill
[3] Department of Obstetrics and Gynecology, University of Wisconsin-Madison
[4] Department of Epidemiology, Gillings School of Global Public Health, University of North Carolina at Chapel Hill
[5] Department of Population Family and Reproductive Health, Johns Hopkins Bloomberg School of Public Health, Baltimore, Maryland, USA.
[6] International Centre for Reproductive Health-Kenya, Nairobi, Kenya
[7] Institut Supérieur des Sciences de la Population (ISSP/University of Ouagadougou), Ouagadougou, Burkina Faso
The following members of the Performance Monitoring for Action (PMA) Principal Investigators group contributed to the data collection and development of this manuscript: Solomon Shiferaw and Assefa Seme, Addis Ababa University, Ethiopia; Elizabeth Omoluabi, Statistics and Population Studies Department, University of the Western Cape, South Africa; Fredrick Edward Makumbi and Simon Kibera, Makerere University School of Public Health, Uganda.

Corresponding Author:
Brooke W. Bullington
Department of Epidemiology
Gillings School of Global Public Health
University of North Carolina at Chapel Hill
bbullington@unc.edu

Key words: Contraceptive coercion, Contraceptive implants, Implant removal, Reproductive justice, long-acting reversible contraception (LARC), sub-Saharan Africa

Word count: 3,493

For peer review only

ABSTRACT

Objectives

Contraceptive implant use has grown considerably in the last decade, particularly among women in Burkina Faso and Kenya, where implant use is among the highest globally. We aim to quantify the proportion of current implant users who have unsuccessfully attempted implant removal in Burkina Faso and Kenya and document reasons for and location of unsuccessful removal.

Methods

We use nationally representative data collected between 2016 and 2020 from women of reproductive age in Burkina Faso and Kenya to estimate the prevalence of implant use, proportion of current implant users who unsuccessfully attempted removal, and proportion of all removal attempts that have been unsuccessful. We describe reasons for and barriers to removal, including the type of facility where successful and unsuccessful attempts occurred.

Findings

Over a four-year period, the percentage of current implant users reporting an unsuccessful removal attempt declined from 9% (95% CI: 7%, 12%) to 2% (95% CI: 1%, 3%) in Kenya and from 7% (95% CI: 4%, 14%) to 3% (95% CI: 2%, 6%) in Burkina Faso. Common barriers to removal included being counseled against removal by the provider or told to return a different day.

Interpretation

Unsuccessful implant removal has decreased in recent years. Despite progress, substantial numbers of women desire having their contraceptive implant removed but are unable to do so. Greater attention to health systems barriers preventing implant removal is imperative to protecting reproductive autonomy and ensuring women can achieve their reproductive goals.

STRENGTHS AND LIMITATIONS

- This analysis uses nationally representative Performance Monitoring for Action data from multiple years across diverse geographies.
- Survey items were asked inconsistently between 2016 and 2020, making direct comparisons difficult in some instances. For example, comparison of reasons and barriers for implant removal was difficult to compare over time.
- Small sample sizes of women seeking implant removal limited greater assessment of unsuccessful removal experiences.
- Data do not capture implant removal experiences of women who switched contraceptive methods.

Background

Access to contraceptive implants has increased substantially in many low- and middle-income countries (LMICs) over the past decade. Political and financial commitments to contraceptive access from governments and international donors has led to increased supply and reduced commodity cost of implants. This has resulted in expanded access to implants in LMICs, diversifying the contraceptive method mix and contributing to substantial growth in the contraceptive prevalence rate, particularly in sub-Saharan Africa. (1,2) Implants, a form of long-acting reversible contraception (LARC), are highly effective, easy to maintain, and relatively low cost over time, making them a popular choice for many women in sub-Saharan Africa. (3,4) In Burkina Faso and Kenya, patterns in contraceptive use have changed dramatically in recent years, as many women have shifted from using injectable contraception to implants. (2,5) In both countries, implants are heavily subsidized by the government and donors, and are provided free of charge or at a low cost. (6,7)

While contraceptive implants offer many advantages, insertion and removal require a trained clinician, posing potential barriers to successful uptake and discontinuation. To uphold principles of human rights, provision of reliable, sustainable, and equitable services for both insertion and removal of implants must be ensured. Lack of access to high-quality implant removal services compromises reproductive autonomy, particularly for individuals who wish to stop using the method. (8) With increased uptake of implants, the demand for removal service in sub-Saharan Africa is estimated to increase substantially in the coming years, yet the extent to which individuals can access such services, as well as barriers they may face in seeking removal, has not been readily researched. (9) Additionally, understanding the unique sociodemographic characteristics of implant users can provide important insights into the potential implications of such barriers.

A 2019 qualitative study shed light on several barriers to person-centered care, specifically for individuals receiving LARC methods. Women from a sub-Saharan African country described being pressured to adopt LARC they did not want or fully understand due to limited choices, biased counselling, scare tactics, and misinformation from service providers. Several women reported being denied removal services before completing the five-year contraceptive efficacy period for the method, despite their desires for removal, even if they wished to become pregnant. (10) Similar findings emerged in a qualitative study from Western Kenya, where women shared experiences of being refused removal and pressured to use LARC for its full duration, regardless of their reported side effects. Other barriers to removal services included high removal costs, both in terms of formal and informal fees. (11) In a mixed-method study among women in Burkina Faso and Uganda, respondents reported anxiety over the potential added costs of method removal. (12) While qualitative evidence demonstrates barriers women face when seeking removal, few quantitative studies have estimated this experience among population-based samples of current and recent implant users, limiting our understanding of the frequency and reasons for unsuccessful removals.

This study seeks to expand upon recent qualitative findings highlighting substantial barriers to implant removal. Using nationally representative data collected between 2016 and 2020 in Burkina Faso and Kenya, we examine sociodemographic characteristics of implant users, recent trends in implant use, unsuccessful removal, and barriers to successful removal. We aim to quantify the proportion of current implant users who have unsuccessfully attempted implant removal in each country, and document reasons for and location of unsuccessful removal over the four-year period.

Methods

Data Source and Sample

This cross-sectional study uses multiple rounds of survey data from the Performance Monitoring for Action (PMA) project, formerly known as PMA2020. Since 2019, PMA has conducted yearly population-based surveys with women of reproductive age (15-49 years), in 10 countries across sub-Saharan Africa and South Asia, generating data on key reproductive health indicators. Data from PMA2020 on implant removal are available in select countries dating back to 2016. We restrict our analysis to the two countries that measured implant removal experiences across multiple rounds of data from PMA2020 and PMA, and for which implants constitute the most widely used method. Based on these criteria, we include data collected in Burkina Faso and Kenya between 2016 and 2020, with each country contributing four rounds of data. Participating women were selected using a multistage sampling strategy. All eligible women were invited to participate via an informed consent process. Enumerators collected data from each participant, including background characteristics, reproductive and contraceptive behaviors, and experiences with contraceptive health services (e.g., implant removal). Additional details of PMA's survey methodology have been published elsewhere and can be found at https://www.pmadata.org/data/survey-methodology. (13)

Patient and Public Involvement

Patients and the public were not involved in the design, conduct, reporting, or dissemination plans of our research.

Measures

Our primary measures assess women's contraceptive use and, among users of the contraceptive implant, experiences of removal. To assess changes in contraceptive use, implant use, and the contraceptive method mix since 2016, we used women's responses to the question, "*Are you or your partner currently doing something or using any method to delay or avoid getting pregnant?*" Among women who reported use, they were asked to specify which method they were currently using. We compare use of the implant to use of other modern methods, defined as female or male sterilization, intrauterine device, injectable contraception, oral contraceptive pill, or male or female condom, emergency contraception, standard days method, or the lactational amenorrhea method. (14,15)

Our main outcome was unsuccessful removal attempt, measured by asking current implant users if they tried to have their current implant removed in the last 12 months (yes/no). Current implant users who reported trying to have their implant removed were categorized as experiencing an "unsuccessful" removal. Our secondary outcomes were reasons for and location of unsuccessful removal, which were ascertained by asking those with unsuccessful removal why they were not able to have their implant removed and where they went or who attempted to remove their implant. Responses to the latter were categorized as a public (i.e., government owned) or private-sector facility/provider.

Additionally, we estimated successful implant removal among women who were not currently using contraception and had used a method in the last 12 months. Current non-users reporting implant use within the last 12 months were classified, de facto, as having

"successful" implant removal[1]. Among women with a successful implant removal in the last 12 months, we also ascertained reasons for – and location of – successful implant removal, also delineating reliance on the public or private sector for this service. In the first three rounds of data included in this analysis (annually, 2016-2019), reason for removal was measured directly by asking women who reported implant removal in the last 12 months *"Why did you stop using your implant?"* For the most recent round of data collection in each country, this question was omitted, and both implant removal and reasons for removal were assessed using data from the contraceptive calendar (restricted to the 12 months preceding the interview). The contraceptive calendar is a retrospective instrument that documents duration of use and reasons for discontinuation, on a month-by-month basis, typically for multiple years.

Notably, not all measures related to implant removal were collected for all years in both countries. The location of successful removal was only captured for two years for each country (Burkina Faso: 2018 and 2019; Kenya: 2017 and 2018) and the location of unsuccessful removal was not collected in Burkina Faso in 2017 or Kenya in 2016. Additionally, reason for unsuccessful implant removal was not collected in Kenya in 2017. Despite these limitations, we use all available data in each geography to examine trends and barriers to removal.

Analysis

We first describe sociodemographic characteristics of modern contraceptive users in each country for the most recent year of data. We use bivariate statistics and Pearson's chi-square tests to explore whether use of the implant versus any other modern method of contraception varied according to women's sociodemographic characteristics. To contextualize trends in access to removal, for each country and survey year, we describe the proportion of women currently using a contraceptive method, those using a modern method, and the overall contraceptive method mix.

Next, we estimate the proportion of current implant users who reported unsuccessful removal. We also estimate the proportion of all implant removal attempts that were unsuccessful by dividing the total number of unsuccessful removal attempts in the past 12 months by all removal attempts in the past 12 months, both successful and unsuccessful. We use descriptive statistics to summarize locations of and reasons for successful and unsuccessful removal. Analyses were computed using Stata 16.0. Survey weights were applied to account for the probability of selection as well as non-response.

Ethical approval for PMA data collection activities was granted by in-country ethics committees in Burkina Faso (Comité d'Ethique pour la Recherche en Santé) and Kenya (Kenya Medical Research Institute (KEMRI) Ethics Review Committee). The Institutional Review Board for human subjects research at the Johns Hopkins Bloomberg School of Public Health reviewed and approved the study protocols for the PMA project.

---

[1] Due to limitations of the existing data (i.e., absence of a contraceptive calendar in select years in both countries), we are unable to estimate the proportion of successful implant removal among *currently* contracepting women (i.e., those women who removed an implant in the last 12 months and were using a different method at the time of data collection); we describe this limitation in further detail in the discussion section.

For peer review only

Results

Table 1 provides background characteristics for all modern contraceptive users in the most recent year of data collection, stratified by use of implants versus other modern methods. Implants users, compared to other modern method users, were more likely to be older, married, less educated, live in rural areas and poorer households, have a greater number of children, and obtain their method from the public sector. While implant users in Kenya were more likely to prefer no more children compared to other modern method users (48% versus 44%, respectively), this difference was small, and no statistically significant differences were seen in the distribution of fertility preferences in Burkina Faso.

Table 1. Characteristics of modern method users compared to implant users, in Burkina Faso and Kenya. 2019.

	Burkina Faso			Kenya		
	2020			2019		
	All Modern	Implant	*p-value	All Modern	Implant	*p-value
**N**	900	778		2463	1575	
**Total % of all women**	14%	12%		26%	17%	
**Age**			0.300			0.0006
15-24	31%	29%		25%	25%	
25-39	55%	50%		55%	61%	
40-49	14%	22%		20%	14%	
**Marital status**			0.000			0.0001
In union	71%	82%		69%	78%	
Sexually active unmarried	29%	18%		31%	22%	
**Parity**			0.000			<0.000
0	17%	4%		11%	1%	
1 to 2	29%	30%		37%	42%	
3 to 4	27%	25%		31%	34%	
5 or more	27%	41%		21%	23%	
**Residence**			0.000			0.0001
Urban	37%	22%		36%	27%	
Rural	63%	78%		64%	73%	
**Education**			0.000			<0.000
No education	46%	64%		2%	4%	
Primary	19%	19%		49%	57%	
Secondary	30%	16%		37%	30%	
Tertiary	4%	1%		13%	9%	
**Wealth tertile**			0.000			<0.000
Lowest	25%	34%		28%	38%	
Middle	28%	34%		37%	36%	
Highest	47%	32%		35%	26%	
**Received from public facility**			0.000			<0.000
Yes	74%	91%		61%	88%	
No	26%	9%		39%	12%	
**Fertility preferences**			0.090			0.0009

For peer review only

No more children	18%	25%	44%	48%
Children in the next 2 years	35%	31%	16%	13%
Children more than 2 years from now	38%	34%	33%	34%
Says she can't get pregnant	1%	1%	2%	1%
Undecided	8%	9%	5%	3%
Other	1%	0%	0%	0%

**We use the chi-square test to assess whether the distribution of characteristics differed based on whether a woman reported implant versus other modern method use.*

In Table 2, we provide important context for trends in implant removal by presenting the prevalence of all contraception, modern contraception, and the method mix in each country for each year of data collection. The total number of participants ranged from 3,221 (2017) to 6,590 (2020) in Burkina Faso and from 5,864 (2017) to 9,469 (2019) in Kenya. In Burkina Faso, contraceptive prevalence increased in the first three years, from 23% in 2017 to 29% in 2019, while contraceptive prevalence remained stable at 45% in Kenya during this time.

Table 2. Contraceptive prevalence and method mix over time, among women of reproductive age in Burkina Faso and Kenya. 2016-2020

	Burkina Faso				Kenya			
	2017	2018	2019	2020	2016	2017	2018	2019
**N**	3221	3512	3329	6590	5891	5864	5658	9469
**Current contraceptive use**								
Yes	23%	28%	29%	27%	45%	45%	46%	45%
No	77%	72%	71%	73%	55%	55%	54%	55%
**Current modern contraceptive use**								
Yes	22%	26%	27%	25%	44%	44%	45%	43%
No	78%	74%	73%	75%	56%	56%	55%	57%
**N**	735	982	960	1800	2659	2592	2566	4271
**Method mix**								
Sterilization	0%	0%	0%	0%	4%	4%	4%	4%
IUD	3%	4%	4%	4%	5%	2%	4%	4%
Implant	43%	45%	39%	43%	28%	32%	34%	36%
Injectable	30%	25%	26%	24%	42%	41%	39%	33%
Pills	11%	11%	10%	9%	8%	8%	7%	7%
Condoms	8%	8%	13%	12%	6%	5%	5%	7%
Standard Days Method	0%	1%	0%	0%	0%	1%	0%	0%
Rhythm	3%	5%	5%	6%	2%	2%	2%	3%
Withdrawal	0%	0%	0%	1%	1%	1%	1%	1%
Emergency contraception	0%	0%	1%	0%	2%	3%	3%	2%
Other modern method	0%	0%	0%	1%	1%	0%	0%	1%
Other	1%	0%	1%	0%	0%	0%	0%	2%

Figures 1a-1b display trends in implant and injectable use. In Burkina Faso, implant use constituted 43% of the method mix in both 2017 (95% CI: 38%, 49%) and 2020 (95% CI: 39%, 48%) but the gap between implants and injectables (the second most common method) grew, as injectable use declined from 30% (95% CI: 23%, 39%) to 24% (95% CI: 19%, 31%). In Kenya, implant use grew from 28% (95% CI: 25%, 30%) in 2016 to 36% (95% CI: 34%, 38%) of the method mix in 2019. Due to a corresponding large decline in injectables (42% to 33%), implants are roughly tied with injectables as the primary contraceptive method in Kenya.

Figure 2 illustrates the percent of current implant users reporting an unsuccessful removal attempt, and Figure 3 presents the percent of all implant removal attempts that have been unsuccessful. In Burkina Faso, the percent of current implant users who reported an unsuccessful removal attempt in the last 12 months declined from 7% (95% CI: 4%, 14%) in 2017 to 3% (95% CI: 2%, 6%) in 2020. In Kenya, the percent of current users reporting unsuccessful removal dropped from 9% (95% CI: 7%, 12%) in 2017 to 2% (95% CI: 1%, 3%) in 2019. Among all removal attempts in Burkina Faso, the percent of removals that were unsuccessful dropped from 28% (95% CI: 19%, 40%) in 2017 to 15% (95% CI: 10%, 22%) in 2020. In contrast, Kenya dropped from 52% (95% CI: 44%, 61%) in 2017 to 13% (95% CI: 9%, 18%) in 2019.

Among women who had successfully removed their implant, we present reasons for removal in Burkina Faso (Figure 4) and Kenya (Figure 5). In Burkina Faso, reasons for removal varied over time, with women in 2016 most frequently reporting removal due to a desire to become pregnant (60%) or concerns about interference with the body's natural process (24%). Although these reasons were still commonly reported in later survey years, the proportion of women reporting these experiences diminished over time. By 2020, concerns over side effects were the primary reason for removal – reported by 42% of women - although more than one in three women still sought removal due to a desire to become pregnant. Method failure (became pregnant) was a frequent reason for removal in 2018 and 2019 but was reported by few women in 2017 and 2020. Very similar patterns were observed in Kenya, with a desire to become pregnant and health concerns/side effects consistently reported by a large percent of respondents across all four rounds of data collection (29-49% and 27-48%, respectively), and up to half (42-51%) reporting method failure in 2017-2018.

Figure 6 presents the location of implant removal and removal attempts, stratified by public versus private sector. Among Burkinabe women, removals were most frequently sourced from a public-sector facility for all years, for both successful removals (75-83%) and unsuccessful removals (85-95%). In Kenya, women sourced successful removal equally from both public and private sector locations in 2017 but far more commonly from the public-sector (79%) in 2018. Like Burkina Faso, most unsuccessful removals in Kenya were sourced from the public sector in all years (71-90%).

Among Burkinabe women who were unsuccessful in obtaining implant removal, self-reported barriers to removal fluctuated from year to year (Figure 7). In 2017, Burkinabe women primarily reported that they were counseled against removal (23%), or a trained provider was unavailable (19%). The following year, women reported a provider refused to remove their implant (18%) or that they could not afford the cost of removal (28%). Cost remained a dominant barrier in 2019 but by 2020, nearly half of women (43%) reported they were counseled against removal, while more than one-third (36%) shared they were told to return to the facility another day. Similarly, in Kenya, common self-reported barriers shifted

For peer review only - http://bmjopen.bmj.com/site/about/guidelines.xhtml

from year to year, with the highest proportion of women in 2016 and 2019 reporting a lack of a trained provider or cost, while in 2018 nearly half of women were counseled against removal or instructed to return another day (Figure 8).

Discussion

In light of growing implant use and recent qualitative research documenting barriers to implant removal in sub-Saharan Africa, we explored changes over time in contraceptive use, implant use, and experiences with implant removal in Burkina Faso and Kenya. Over a four-year period, contraceptive prevalence was relatively steady in both settings. As a percentage of the overall method mix, implant use in Burkina Faso did not vary substantially from 2017 to 2020 (remaining around 43%) while injectable use occupied an *increasingly* distant second place. In contrast, Kenya experienced a steady increase in implant use, coupled with commensurate declines in injectable use.

Given the predominance of implants in both settings, access to method removal is imperative. We found that the percent of current implant users reporting an unsuccessful removal attempt declined in both countries. Reasons for this trend are unclear. Given more than one out of every 10 women of reproductive age in each country is currently using a contraceptive implant, the 2% to 3% of current users unable to remove their implant translates into tens of thousands of women who experience structural forms of contraceptive coercion that prevent them from achieving their contraceptive goals.

In both Burkina Faso and Kenya, more than one in eight women seeking implant removal were unsuccessful in their attempt. In Kenya, this is evidence of a stark downward trend from one in two women just three years prior – suggesting considerable progress has been made to help reduce barriers to removal in Kenya; yet more progress is needed. The most common reasons women seek removal changed over time in both countries; however, wanting to get pregnant or becoming pregnant and concerns about the implant's effect on the body and side effects were persistent reasons for desired removal in both countries. When women provided reasons for unsuccessful implant removals, facility-level barriers were common, including being counselled against removal, told a trained provider is unavailable, or asked to return another day. Cost was also a persistent barrier across time in both countries. Our results confirm prior findings that women encounter substantial facility-level barriers to implant removal as well as unaffordable removal fees, including within public facilities. (10,11,16–18)

Contraceptive implant users – compared to all other modern method users - were consistently older and more likely to be married, which may confer some degree of agency. However, they were also more likely to be uneducated, poor, and living in rural locations, where method removal may rely heavily on public-sector facilities. Women in both countries largely source implant removal – both successful and unsuccessful – from the public sector, where a nascent body of literature suggests providers are resistant to implant removal - at times to preserve limited implant supplies or sometimes due to an aversion for the procedure itself. Additionally, when noting the combination of cost as a substantial barrier and public facilities as the source, we posit a system of substantial informal fees for implant removal may enable providers to avoid removing implants, particularly among poor public-sector family planning clients who are especially price-sensitive. (11,19)

For peer review only

This analysis benefits from multiple years of nationally representative data collected in diverse geographies. However, we acknowledge several limitations. First, we are limited by inconsistent survey items across all years of data. For example, in some years and for questions related to reasons for (or barriers to) implant removal, multiple responses were allowed, while in other years a single response option was used. This makes direct comparison of the frequency of reasons and barriers over time more difficult, as women may have multiple reasons for seeking or failing to have an implant removed but would have been unable to report all reasons in select survey years, resulting in under-reporting of secondary reasons. Similarly, the change in survey structure to include the contraceptive calendar in the most recent round of data collection resulted in minor differences in how women were asked about implant removal as well as under-reporting of implant discontinuation in the years preceding the calendar, during which only non-contraceptive users were asked about prior method use in the last 12 months. Second, small sample sizes for some of the analyses limited greater assessment of unsuccessful removal experiences. Third, among those with successful implant removal in the year prior to the survey, we were unable to measure whether these women first experienced a barrier to removal prior to successful removal. And, among those who were unsuccessful, we cannot report on the total number of unsuccessful attempts. This may have resulted in an underestimate of the percentage of current users that have experienced barriers to removal, presenting a conservative picture of the difficulty of implant removal. Finally, these data do not capture implant removal experience of contraceptive switchers. Ideally, those women who switched from the implant to another method should be classified as having a successful removal, but current users were not asked about prior use in survey rounds without a calendar. As such, for select years we may have underestimated the number of successful removals, which would lead to an overestimation of the proportion of all removals that were unsuccessful.

Despite these limitations, our findings are in line with those in other studies that have examined implant removal in sub-Saharan Africa. In a study following LARC users over one year, 5% of implant users in Nigeria and 7% of implant users in Zambia reported wanting their implants removed, but still using their method. (20) Another study in Ghana noted that only 55-61% of implant users who attempted removal successfully obtained removal on their first attempt. (21) These studies also found similar reasons for wanting implants removed, including side effects and desire for more children, and similar reasons for implant removal failure, including provider counselling to continue using the method. (20,21)

This analysis provides an important contribution to the literature on barriers to implant removal in sub-Saharan Africa. Using nationally representative data to assess changes over time in barriers to removal across two distinct countries, we note both much needed reductions in these barriers and also highlight the additional progress required to sufficiently safeguard contraceptive autonomy in LMICs. Our findings contribute to a growing body of literature highlighting the imperative for improved, patient-centered implant removal services in sub-Saharan Africa. (10,11,20,21) Despite international calls to ensure programmatic support for implant removal services scaled appropriately with the popularity of the implant, barriers to implant removal services persist. (2,9) Family planning programs and services must prioritize contraceptive autonomy, respecting a person's right to choose to discontinue their method at any time and for any reason.

Author Contributions

Data analysis was conceived by KT, LS, and CK, all of whom contributed to the analysis plan and paper framing. Quantitative analysis was conducted by BB and KT, with some input

For peer review only

from LS and CK. KT, BB, SC, and EG all drafted portions of the initial manuscript. CK, LZ, PG, MT, GG, and the PMA PI Group contributed to data collection, data curation, data cleaning, and project administration. All authors contributed substantially to review and revision of the manuscript and have read and approved the final manuscript.

Declaration of interests
We declare no competing interest.

Data sharing
Access to the deidentified datasets used in this analysis are granted on a per request basis by Performance Monitoring and Action. To request datasets, please go to: https://www.pmadata.org/data/request-access-datasets

Ethical approval and consent to participate
Ethical approval for PMA data collection activities has been granted by in-country ethics committees in Burkina Faso (Comité d'Ethique pour la Recherche en Santé, #A14-2020) and Kenya (Kenya Medical Research Institute (KEMRI) Ethics Review Committee, #P241/04/2020). The Institutional Review Board for human subjects research at the Johns Hopkins Bloomberg School of Public Health reviewed and approved the study protocols for the PMA project.

Funding and Acknowledgements
This work was supported, in part, by the Bill & Melinda Gates Foundation IVN 009639. Under the grant conditions of the Foundation, a Creative Commons Attribution 4.0 Generic License has already been assigned to the Author Accepted Manuscript version that might arise from this submission. Support for this research was also provided, in part, by a career development grant (R00 HD086270) to K. Tumlinson and an infrastructure grant for population research (P2C HD050924) to the Carolina Population Center at the University of North Carolina at Chapel Hill, both from The Eunice Kennedy Shriver National Institute of Child Health and Human Development (NICHD) of the National Institutes of Health (NIH). LS's contribution was supported by a Ruth L Kirschstein National Research Service Award (T32 HD049302) and Population Research Infrastructure grant (P2C HD047873) from the NICHD. BB's contribution was supported in part by funding from NICHD (T32 HD52468) and Population Infrastructure grant (P2C HD050924). Contributions by CK, LZ, PG, MT, GG, and the PMA PI Group were supported by grants OPP1198333 and OPP1198339 awarded by the Bill & Melinda Gates Foundation. We wish to gratefully acknowledge the support of Matt Gunther, research staff with IPUMS, for extracting and harmonizing several key variables from the PMA retrospective reproductive calendar data in Kenya and Burkina Faso. The contents of this article are solely the responsibility of the authors and do not necessarily represent the official views of the NIH/NICHD or the Bill & Melinda Gates Foundation.

The PMA project relies on the work of many individuals, both in the United States and in survey countries. The project team is grateful for support from the Bill & Melinda Gates Foundation and would like to thank the country teams and resident enumerators who are ultimately responsible for the success of PMA.

References

For peer review only

1. Tumlinson K, Steiner MJ, Rademacher KH, Olawo A, Solomon M, Bratt J. The promise of affordable implants: is cost recovery possible in Kenya? Contraception. 2011 Jan;83(1):88–93.

2. Jacobstein R. Liftoff: the blossoming of contraceptive implant use in africa. Glob Health Sci Pract. 2018 Mar 21;6(1):17–39.

3. Winner B, Peipert JF, Zhao Q, Buckel C, Madden T, Allsworth JE, et al. Effectiveness of Long-Acting Reversible Contraception. Obstet Gynecol Surv. 2012 Sep;67(9):552–3.

4. Bahamondes L, Fernandes A, Monteiro I, Bahamondes MV. Long-acting reversible contraceptive (LARCs) methods. Best Pract Res Clin Obstet Gynaecol. 2020 Jul;66:28–40.

5. Kungu W, Khasakhala A, Agwanda A. Use of long-acting reversible contraception among adolescents and young women in Kenya. PLoS ONE. 2020 Nov 10;15(11):e0241506.

6. Kiemtoré S, Zoungrana Z, Zamané H, Kaboré CWPD, Ouédraogo A, Bonané B. Interventions to improve the use of long-acting reversible contraceptive methods at primary health centers in Burkina Faso. Int J Gynaecol Obstet. 2019 Dec;147(3):350–5.

7. Radovich E, Dennis ML, Barasa E, Cavallaro FL, Wong KL, Borghi J, et al. Who pays and how much? A cross-sectional study of out-of-pocket payment for modern contraception in Kenya. BMJ Open. 2019 Feb 20;9(2):e022414.

8. Ensuring human rights in the provision of contraceptive information and services: guidance and recommendations. Geneva: World Health Organization; 2014.

9. Christofield M, Lacoste M. Accessible Contraceptive Implant Removal Services: An Essential Element of Quality Service Delivery and Scale-Up. Glob Health Sci Pract. 2016 Sep 28;4(3):366–72.

10. Senderowicz L. "I was obligated to accept": A qualitative exploration of contraceptive coercion. Soc Sci Med. 2019 Oct;239:112531.

11. Britton LE, Williams CR, Onyango D, Wambua D, Tumlinson K. "When it comes to time of removal, nothing is straightforward": A qualitative study of experiences with barriers to removal of long-acting reversible contraception in Western Kenya. Contraception: X. 2021 Mar 22;3:100063.

12. Brunie A, Callahan RL, Mackenzie A, Kibira SPS, Wayack-Pambè M. Developing acceptable contraceptive methods: Mixed-method findings on preferred method characteristics from Burkina Faso and Uganda. [version 2; peer review: 2 approved]. Gates Open Res. 2019 Apr 11;3:1205.

For peer review only

13. Zimmerman L, Olson H, PMA2020 Principal Investigators Group, Tsui A, Radloff S. PMA2020: Rapid Turn-Around Survey Data to Monitor Family Planning Service and Practice in Ten Countries. Stud Fam Plann. 2017 Aug 28;48(3):293–303.

14. World Health Organization Department of Reproductive Health and Research (WHO/RHR) and Johns Hopkins Bloomberg School of Public Health/Center for Communication Programs (CCP), Knowledge for Health Project. Family Planning: A Global Handbook for Providers (2018 Update). Updated 3rd. Baltimore and Geneva: CCP and WHO; 2021.

15. Festin MPR, Kiarie J, Solo J, Spieler J, Malarcher S, Van Look PFA, et al. Moving towards the goals of FP2020 - classifying contraceptives. Contraception. 2016 Oct;94(4):289–94.

16. Yirgu R, Wood SN, Karp C, Tsui A, Moreau C. “You better use the safer one… leave this one”: the role of health providers in women’s pursuit of their preferred family planning methods. BMC Womens Health. 2020 Aug 12;20(1):170.

17. Senderowicz L, Kolenda A. “She told me no, that you cannot change”: Understanding provider refusal to remove contraceptive implants. SSM - Qualitative Research in Health. 2022 Dec;2:100154.

18. Senderowicz L, Karp C, Bullington BW, Tumlinson K, Zimmerman L, OlaOlorun FM, et al. Facility readiness to remove subdermal contraceptive implants in 6 sub-Saharan African countries. AJOG Glob Rep. 2022 Nov 5;2(4):100132.

19. Tumlinson K, Britton LE, Williams CR, Wambua DM, Otieno Onyango D. Informal payments for family planning: prevalence and perspectives of women, providers, and health sector key informants in western Kenya. Sexual and Reproductive Health Matters. 2021 Dec;29(1):1–17.

20. Brunie A, Stankevitz K, Nwala AA, Nqumayo M, Chen M, Danna K, et al. Expanding long-acting contraceptive options: a prospective cohort study of the hormonal intrauterine device, copper intrauterine device, and implants in Nigeria and Zambia. Lancet Glob Health. 2021 Oct;9(10):e1431–41.

21. Callahan R, Lebetkin E, Brennan C, Kuffour E, Boateng A, Tagoe S, et al. What Goes In Must Come Out: A Mixed-Method Study of Access to Contraceptive Implant Removal Services in Ghana. Glob Health Sci Pract. 2020 Jun 30;8(2):220–38.

For peer review only

Figure 1a: Implants and injectables as a percent of the total modern contraceptive method mix, Burkina Faso

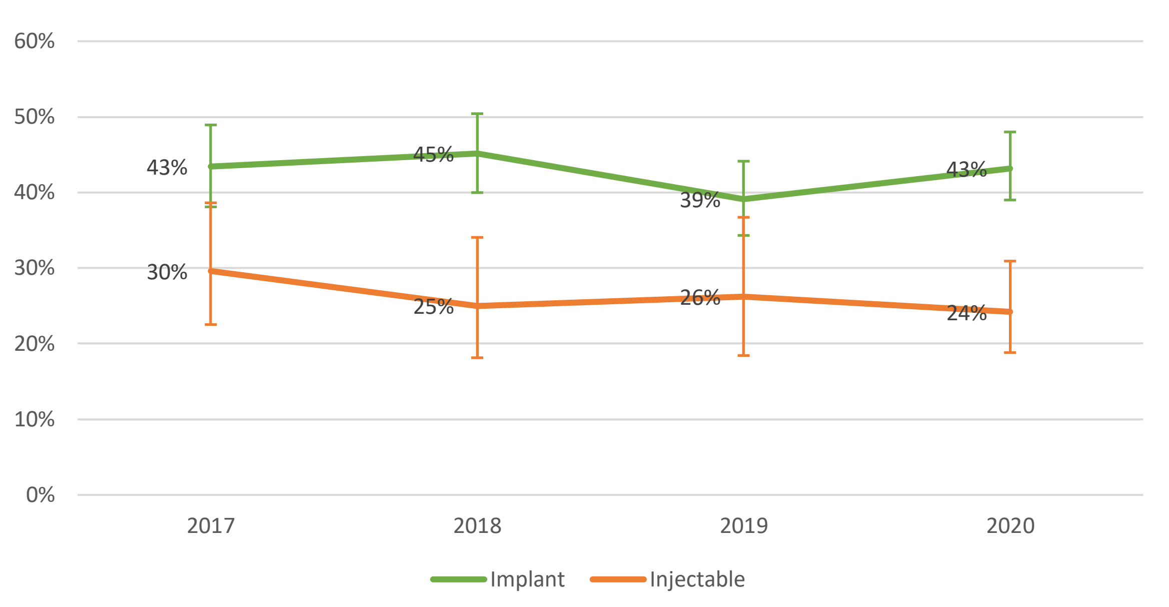

Figure 1b: Implants and injectables as a percent of the total modern contraceptive method mix, Kenya

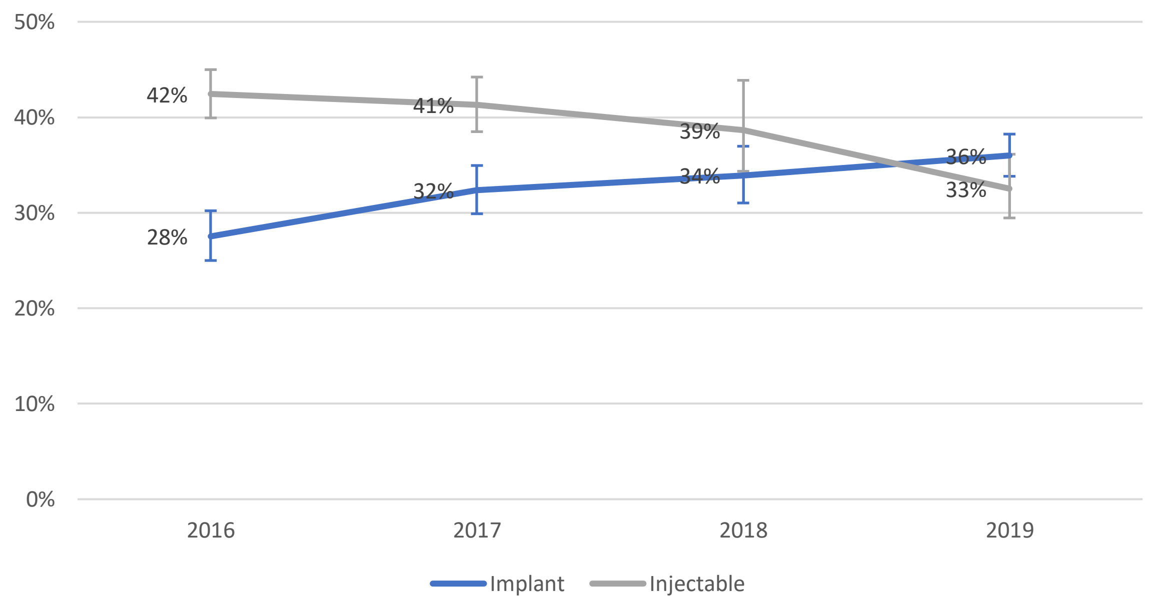

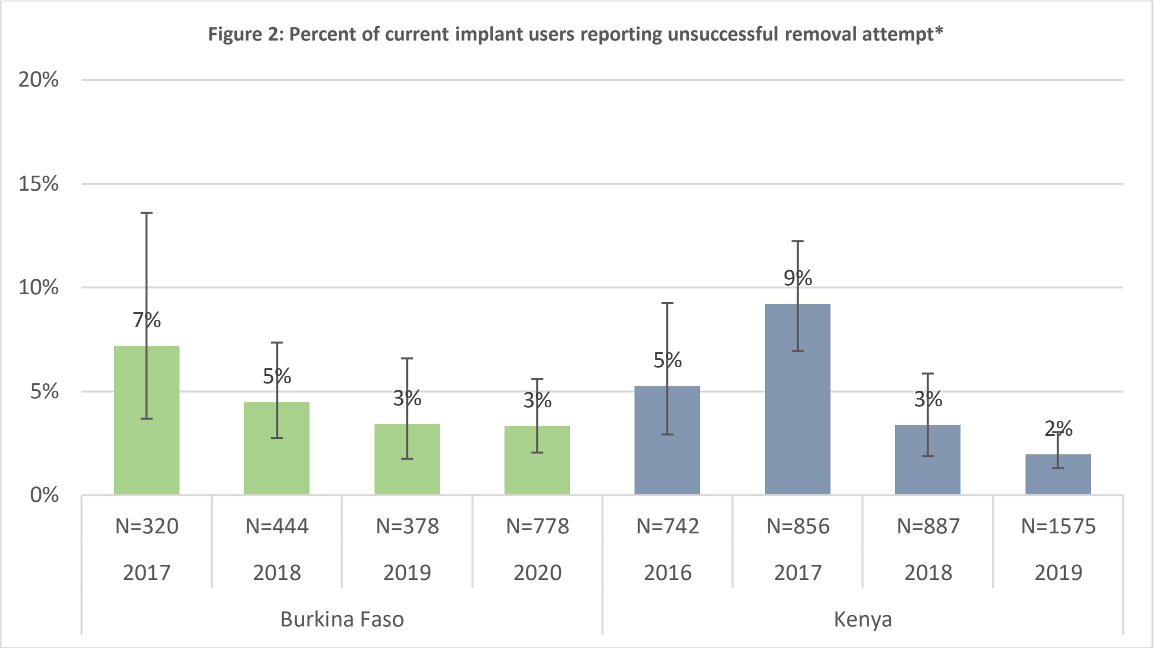

*Denominator is all current implant users.

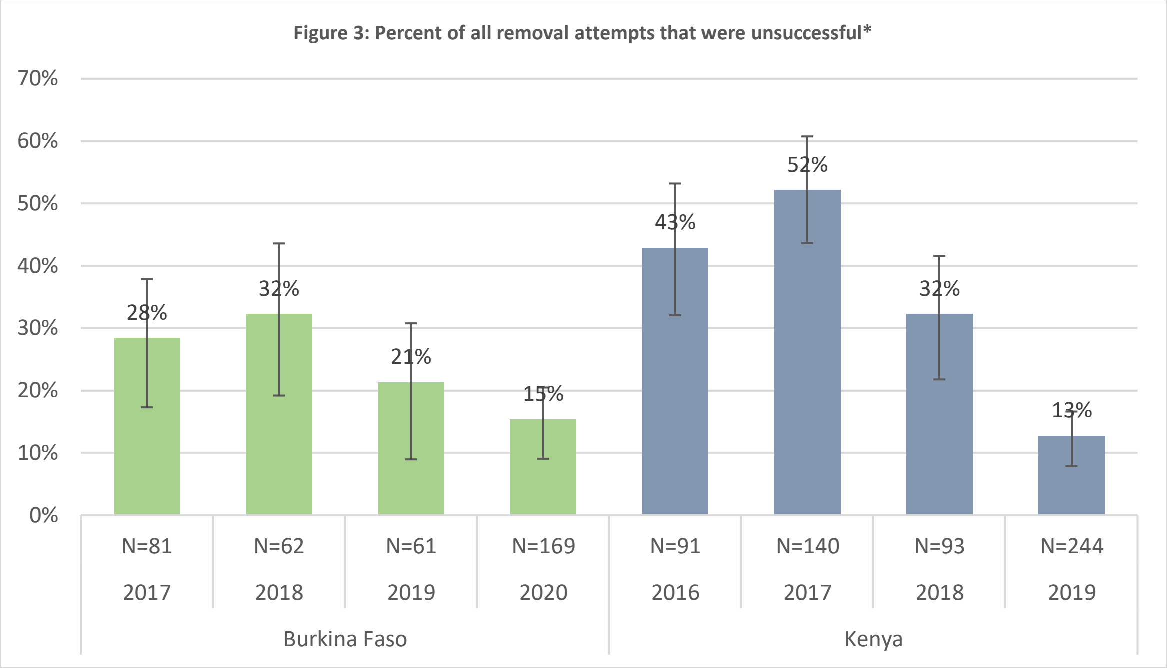

*Denominator is women with successful implant removal in the last 12 months and women with unsuccessful implant removal in the last 12 months.

For peer review only - http://bmjopen.bmj.com/site/about/guidelines.xhtml

Figure 4: Reasons for implant removal, Burkina Faso*

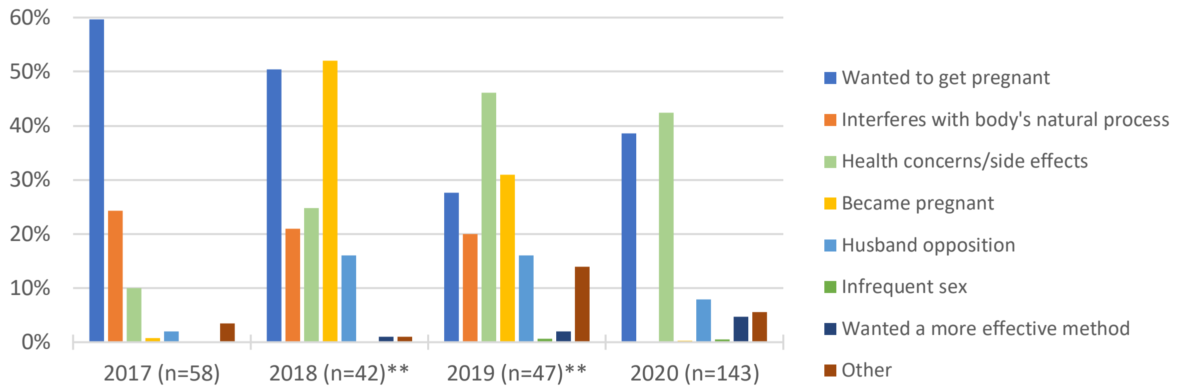

***results may not sum to 100% as survey allowed for multiple responses*

For peer review only

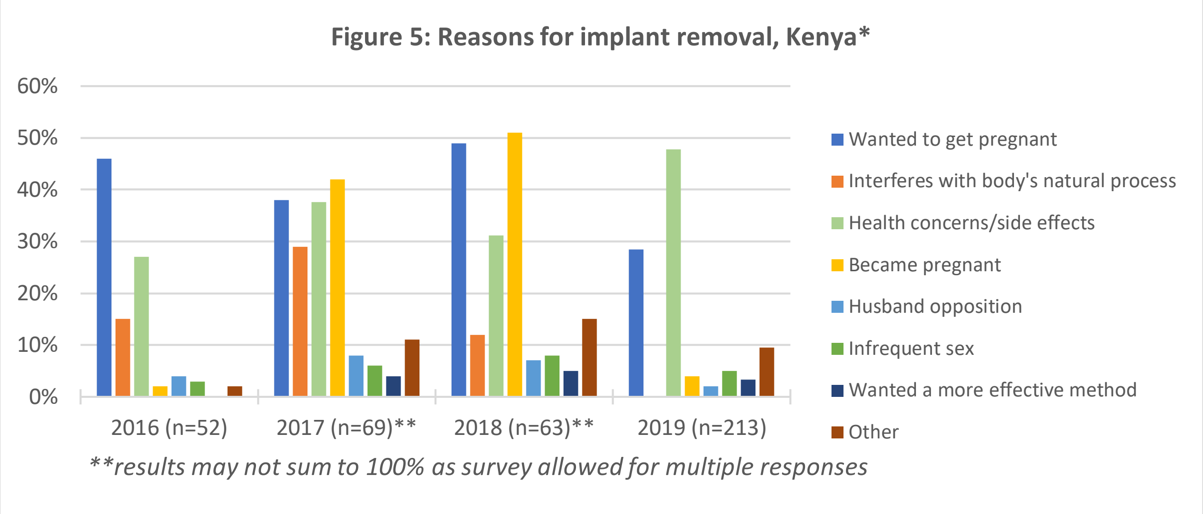

For peer review only

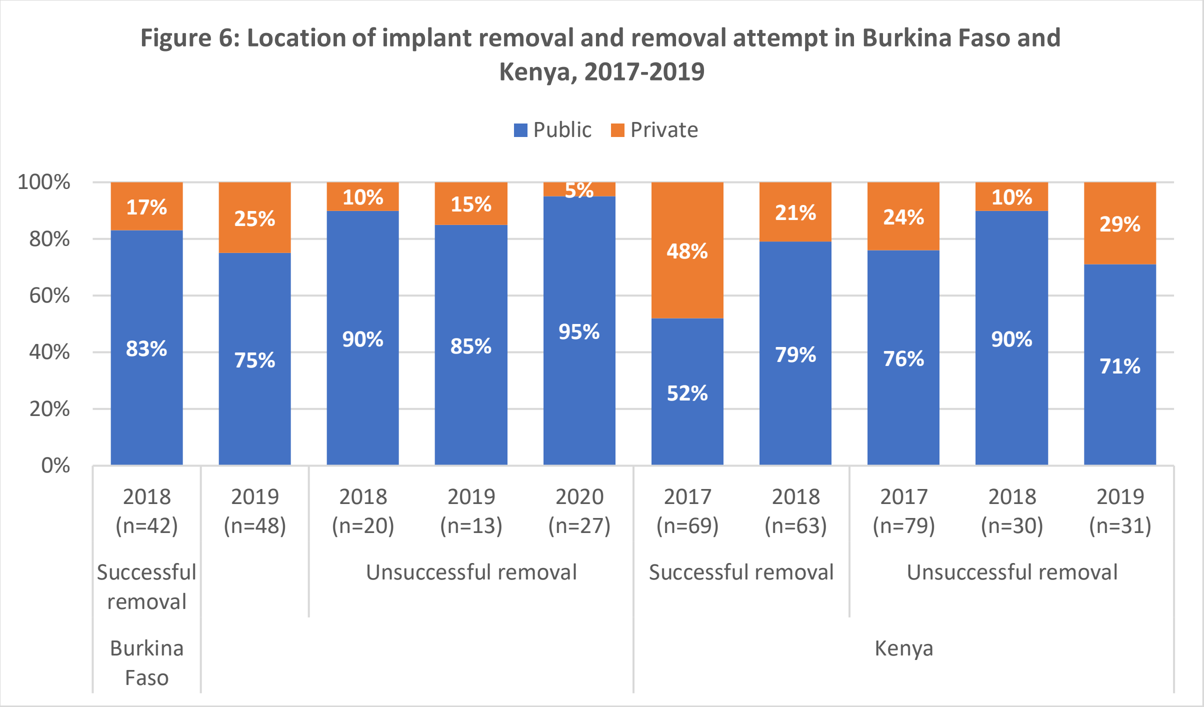

For peer review only

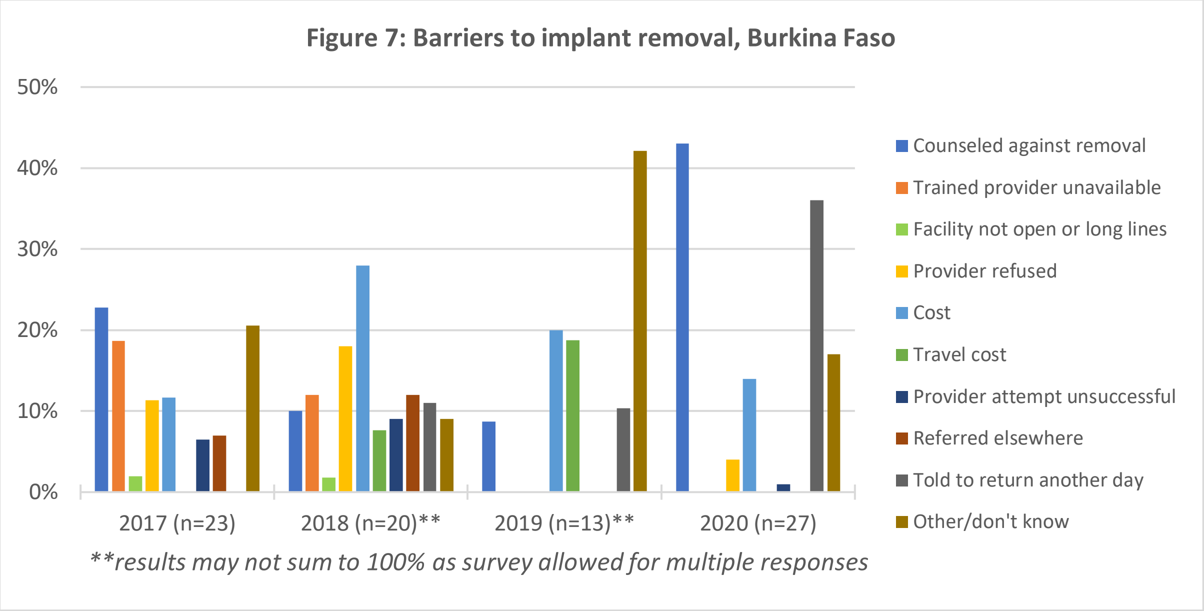

peer review only

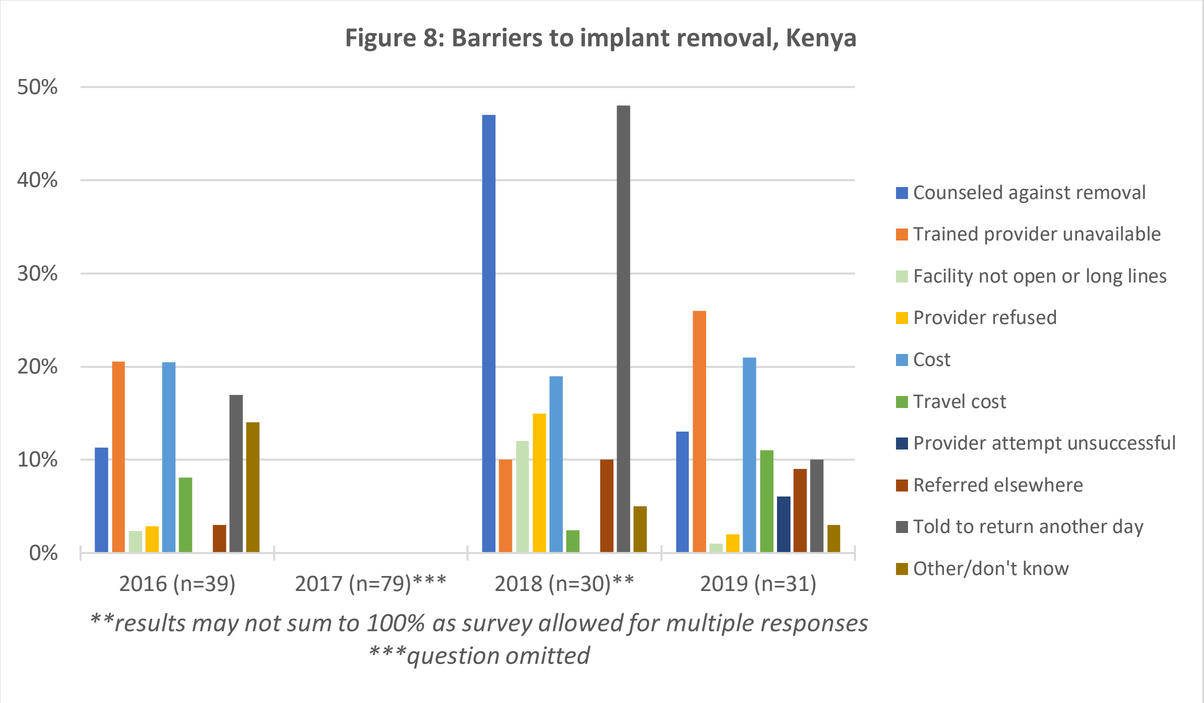

For peer review only

STROBE 2007 (v4) Statement—Checklist of items that should be included in reports of *cross-sectional studies*

Section/Topic	Item #	Recommendation	Reported on page #
**Title and abstract**	1	(*a*) Indicate the study's design with a commonly used term in the title or the abstract	2
		(*b*) Provide in the abstract an informative and balanced summary of what was done and what was found	2
**Introduction**			
Background/rationale	2	Explain the scientific background and rationale for the investigation being reported	3
Objectives	3	State specific objectives, including any prespecified hypotheses	3
**Methods**			
Study design	4	Present key elements of study design early in the paper	4
Setting	5	Describe the setting, locations, and relevant dates, including periods of recruitment, exposure, follow-up, and data collection	4
Participants	6	(*a*) Give the eligibility criteria, and the sources and methods of selection of participants	4
Variables	7	Clearly define all outcomes, exposures, predictors, potential confounders, and effect modifiers. Give diagnostic criteria, if applicable	4-5
Data sources/ measurement	8*	For each variable of interest, give sources of data and details of methods of assessment (measurement). Describe comparability of assessment methods if there is more than one group	4-5
Bias	9	Describe any efforts to address potential sources of bias	*4-5*
Study size	10	Explain how the study size was arrived at	6
Quantitative variables	11	Explain how quantitative variables were handled in the analyses. If applicable, describe which groupings were chosen and why	4-5
Statistical methods	12	(*a*) Describe all statistical methods, including those used to control for confounding	5
		(*b*) Describe any methods used to examine subgroups and interactions	NA
		(*c*) Explain how missing data were addressed	4-5
		(*d*) If applicable, describe analytical methods taking account of sampling strategy	NA
		(*e*) Describe any sensitivity analyses	NA
**Results**			

For peer review only

Participants	13*	(a) Report numbers of individuals at each stage of study—eg numbers potentially eligible, examined for eligibility, confirmed eligible, included in the study, completing follow-up, and analysed	6
		(b) Give reasons for non-participation at each stage	6
		(c) Consider use of a flow diagram	NA
Descriptive data	14*	(a) Give characteristics of study participants (eg demographic, clinical, social) and information on exposures and potential confounders	6
		(b) Indicate number of participants with missing data for each variable of interest	6
Outcome data	15*	Report numbers of outcome events or summary measures	6-7
Main results	16	(*a*) Give unadjusted estimates and, if applicable, confounder-adjusted estimates and their precision (eg, 95% confidence interval). Make clear which confounders were adjusted for and why they were included	6-7
		(*b*) Report category boundaries when continuous variables were categorized	4-5
		(*c*) If relevant, consider translating estimates of relative risk into absolute risk for a meaningful time period	NA
Other analyses	17	Report other analyses done—eg analyses of subgroups and interactions, and sensitivity analyses	NA
**Discussion**			
Key results	18	Summarise key results with reference to study objectives	7
Limitations	19	Discuss limitations of the study, taking into account sources of potential bias or imprecision. Discuss both direction and magnitude of any potential bias	8
Interpretation	20	Give a cautious overall interpretation of results considering objectives, limitations, multiplicity of analyses, results from similar studies, and other relevant evidence	7-8
Generalisability	21	Discuss the generalisability (external validity) of the study results	7-8
**Other information**			
Funding	22	Give the source of funding and the role of the funders for the present study and, if applicable, for the original study on which the present article is based	9

*Give information separately for cases and controls in case-control studies and, if applicable, for exposed and unexposed groups in cohort and cross-sectional studies.

Note: An Explanation and Elaboration article discusses each checklist item and gives methodological background and published examples of transparent reporting. The STROBE checklist is best used in conjunction with this article (freely available on the Web sites of PLoS Medicine at http://www.plosmedicine.org/, Annals of Internal Medicine at http://www.annals.org/, and Epidemiology at http://www.epidem.com/). Information on the STROBE Initiative is available at www.strobe-statement.org.

For peer review only

Assessing trends and reasons for unsuccessful implant discontinuation in Burkina Faso and Kenya between 2016 and 2020: A cross-sectional study

Journal:	*BMJ Open*
Manuscript ID	bmjopen-2023-071775.R1
Article Type:	Original research
Date Submitted by the Author:	11-Apr-2023
Complete List of Authors:	Tumlinson, Katherine; The University of North Carolina at Chapel Hill, Department of Maternal and Child Health<br>Senderowicz, Leigh ; University of Wisconsin-Madison, Department of Gender and Women's Studies<br>Bullington, Brooke; The University of North Carolina at Chapel Hill, Department of Epidemiology<br>Chung, Stephanie; The University of North Carolina at Chapel Hill, Department of Maternal and Child Health<br>Goland, Emilia; The University of North Carolina at Chapel Hill, Department of Maternal and Child Health<br>Zimmerman, Linnea; Johns Hopkins University Bloomberg School of Public Health, Department of Population, Family & Reproductive Health, Johns Hopkins Bloomberg School of Public Health, Baltimore, MD, USA<br>Gichangi, Peter; International Centre for Reproductive Health Kenya; University of Nairobi<br>Thiongo, Mary; International Centre for Reproductive Health Kenya<br>Guiella, Georges; ISSP/University of Ouagadougou)<br>NA, PMA Principal Investigators Group; NA<br>Karp, Celia; Johns Hopkins University Bloomberg School of Public Health
**Primary Subject Heading**:	Obstetrics and gynaecology
Secondary Subject Heading:	Public health, Reproductive medicine
Keywords:	REPRODUCTIVE MEDICINE, PUBLIC HEALTH, EPIDEMIOLOGY, Quality in health care < HEALTH SERVICES ADMINISTRATION & MANAGEMENT

I, the Submitting Author has the right to grant and does grant on behalf of all authors of the Work (as defined in the below author licence), an exclusive licence and/or a non-exclusive licence for contributions from authors who are: i) UK Crown employees; ii) where BMJ has agreed a CC-BY licence shall apply, and/or iii) in accordance with the terms applicable for US Federal Government officers or employees acting as part of their official duties; on a worldwide, perpetual, irrevocable, royalty-free basis to BMJ Publishing Group Ltd ("BMJ") its licensees and where the relevant Journal is co-owned by BMJ to the co-owners of the Journal, to publish the Work in this journal and any other BMJ products and to exploit all rights, as set out in our licence.

The Submitting Author accepts and understands that any supply made under these terms is made by BMJ to the Submitting Author unless you are acting as an employee on behalf of your employer or a postgraduate student of an affiliated institution which is paying any applicable article publishing charge ("APC") for Open Access articles. Where the Submitting Author wishes to make the Work available on an Open Access basis (and intends to pay the relevant APC), the terms of reuse of such Open Access shall be governed by a Creative Commons licence – details of these licences and which Creative Commons licence will apply to this Work are set out in our licence referred to above.

Other than as permitted in any relevant BMJ Author's Self Archiving Policies, I confirm this Work has not been accepted for publication elsewhere, is not being considered for publication elsewhere and does not duplicate material already published. I confirm all authors consent to publication of this Work and authorise the granting of this licence.

For peer review only

Title: Assessing trends and reasons for unsuccessful implant discontinuation in Burkina Faso and Kenya between 2016 and 2020: A cross-sectional study

Authors: Katherine Tumlinson[1,2], Leigh Senderowicz[3], Brooke W. Bullington[2,4], Stephanie Chung[1], Emilia Goland[1], Linnea Zimmerman[5], Peter Gichangi[6], Mary Thiongo[6], Georges Guiella[7], PMA Principal Investigators Group[#], Celia Karp[5]

Affiliations:
[1] Department of Maternal and Child Health, Gillings School of Global Public Health, University of North Carolina at Chapel Hill
[2] Carolina Population Center, University of North Carolina at Chapel Hill
[3] Department of Obstetrics and Gynecology, University of Wisconsin-Madison
[4] Department of Epidemiology, Gillings School of Global Public Health, University of North Carolina at Chapel Hill
[5] Department of Population Family and Reproductive Health, Johns Hopkins Bloomberg School of Public Health, Baltimore, Maryland, USA.
[6] International Centre for Reproductive Health-Kenya, Nairobi, Kenya
[7] Institut Supérieur des Sciences de la Population (ISSP/University of Ouagadougou), Ouagadougou, Burkina Faso
The following members of the Performance Monitoring for Action (PMA) Principal Investigators group contributed to the data collection and development of this manuscript: Solomon Shiferaw and Assefa Seme, Addis Ababa University, Ethiopia; Elizabeth Omoluabi, Statistics and Population Studies Department, University of the Western Cape, South Africa; Fredrick Edward Makumbi and Simon Kibera, Makerere University School of Public Health, Uganda.

Corresponding Author:
Brooke W. Bullington
Department of Epidemiology
Gillings School of Global Public Health
University of North Carolina at Chapel Hill
bbullington@unc.edu

Key words: Contraceptive coercion, Contraceptive implants, Implant removal, Reproductive justice, long-acting reversible contraception (LARC), sub-Saharan Africa

Word count: 3,493

For peer review only

ABSTRACT

Objectives

Contraceptive implant use has grown considerably in the last decade, particularly among women in Burkina Faso and Kenya, where implant use is among the highest globally. We aim to quantify the proportion of current implant users who have unsuccessfully attempted implant removal in Burkina Faso and Kenya and document reasons for and location of unsuccessful removal.

Methods

We use nationally representative data collected between 2016 and 2020 from a cross-section of women of reproductive age in Burkina Faso and Kenya to estimate the prevalence of implant use, proportion of current implant users who unsuccessfully attempted removal, and proportion of all removal attempts that have been unsuccessful. We describe reasons for and barriers to removal, including the type of facility where successful and unsuccessful attempts occurred.

Findings

The total number of participants ranged from 3,221 (2017) to 6,590 (2020) in Burkina Faso and from 5,864 (2017) to 9,469 (2019) in Kenya. Over a four-year period, the percentage of current implant users reporting an unsuccessful implant discontinuation declined from 9% (95% CI: 7%, 12%) to 2% (95% CI: 1%, 3%) in Kenya and from 7% (95% CI: 4%, 14%) to 3% (95% CI: 2%, 6%) in Burkina Faso. Common barriers to removal included being counseled against removal by the provider or told to return a different day.

Conclusion

Unsuccessful implant discontinuation has decreased in recent years. Despite progress, substantial numbers of women desire having their contraceptive implant removed but are unable to do so. Greater attention to health systems barriers preventing implant removal is imperative to protecting reproductive autonomy and ensuring women can achieve their reproductive goals.

STRENGTHS AND LIMITATIONS

- This analysis uses nationally representative Performance Monitoring for Action data from multiple years across diverse geographies.
- Survey items were asked inconsistently between 2016 and 2020, making direct comparisons difficult in some instances. For example, comparison of reasons and barriers for implant removal was difficult to compare over time.
- Small sample sizes of women seeking implant removal limited greater assessment of unsuccessful removal experiences.
- Data do not capture implant removal experiences of women who switched contraceptive methods.

Background

Access to contraceptive implants has increased substantially in many low- and middle-income countries (LMICs) over the past decade. Political and financial commitments to contraceptive access from governments and international donors has led to increased supply and reduced commodity cost of implants. This has resulted in expanded access to implants in LMICs, diversifying the contraceptive method mix and contributing to substantial growth in the contraceptive prevalence rate, particularly in sub-Saharan Africa. (1,2) Implants, a form of long-acting reversible contraception (LARC), are highly effective, easy to maintain, and relatively low cost over time, making them a popular choice for many women in sub-Saharan Africa. (3,4) In Burkina Faso and Kenya, patterns in contraceptive use have changed dramatically in recent years, as many women have shifted from using injectable contraception to implants. (2,5) In both countries, implants are heavily subsidized by the government and donors, and are provided free of charge or at a low cost. (6,7)

While contraceptive implants offer many advantages, insertion and removal require a trained clinician, posing potential barriers to successful uptake and discontinuation. To uphold principles of human rights, provision of reliable, sustainable, and equitable services for both insertion and removal of implants must be ensured. Lack of access to high-quality implant removal services compromises reproductive autonomy, particularly for individuals who wish to stop using the method. (8) With increased uptake of implants, the demand for removal service in sub-Saharan Africa is estimated to increase substantially in the coming years, yet the extent to which individuals can access such services, as well as barriers they may face in seeking removal, has not been readily researched. (9) Additionally, understanding the unique sociodemographic characteristics of implant users can provide important insights into the potential implications of such barriers.

A 2019 qualitative study shed light on several barriers to person-centered care, specifically for individuals receiving LARC methods. Women from a sub-Saharan African country described being pressured to adopt LARC they did not want or fully understand due to limited choices, biased counselling, scare tactics, and misinformation from service providers. Several women reported being denied removal services before completing the five-year contraceptive efficacy period for the method, despite their desires for removal, even if they wished to become pregnant. (10) Similar findings emerged in a qualitative study from Western Kenya, where women shared experiences of being refused removal and pressured to use LARC for its full duration, regardless of their reported side effects. Other barriers to removal services included high removal costs, both in terms of formal and informal fees. (11) In a mixed-method study among women in Burkina Faso and Uganda, respondents reported anxiety over the potential added costs of method removal. (12) While qualitative evidence demonstrates barriers women face when seeking removal, few quantitative studies have estimated this experience among population-based samples of current and recent implant users, limiting our understanding of the frequency and reasons for unsuccessful removals.

This study seeks to expand upon recent qualitative findings highlighting substantial barriers to implant removal. Using nationally representative data collected between 2016 and 2020 in Burkina Faso and Kenya, we examine sociodemographic characteristics of implant users, recent trends in implant use, unsuccessful removal, and barriers to successful removal. We aim to quantify the proportion of current implant users who have unsuccessfully attempted implant removal in each country, and document reasons for and location of unsuccessful removal over the four-year period.

For peer review only

Methods

Data Source and Sample

This cross-sectional study uses multiple rounds of survey data from the Performance Monitoring for Action (PMA) project, formerly known as PMA2020. Since 2019, PMA has conducted yearly population-based surveys with women of reproductive age (15-49 years), in 10 countries across sub-Saharan Africa and South Asia, generating data on key reproductive health indicators. Data from PMA2020 on implant removal are available in select countries dating back to 2016. We restrict our analysis to the two countries that measured implant removal experiences across multiple rounds of data from PMA2020 and PMA, and for which implants constitute the most widely used method. Based on these criteria, we include data collected in Burkina Faso and Kenya between 2016 and 2020, with each country contributing four rounds of data. Participating women were selected using a multistage sampling strategy. All eligible women were invited to participate via an informed consent process. Enumerators collected data from each participant, including background characteristics, reproductive and contraceptive behaviors, and experiences with contraceptive health services (e.g., implant removal). Additional details of PMA's survey methodology have been published elsewhere and can be found at https://www.pmadata.org/data/survey-methodology. (13)

Patient and Public Involvement

Patients and the public were not involved in the design, conduct, reporting, or dissemination plans of our research.

Measures

Our primary measures assess women's contraceptive use and, among users of the contraceptive implant, experiences of removal. To assess changes in contraceptive use, implant use, and the contraceptive method mix since 2016, we used women's responses to the question, "*Are you or your partner currently doing something or using any method to delay or avoid getting pregnant?*" Among women who reported use, they were asked to specify which method they were currently using. We compare use of the implant to use of other modern methods, defined as female or male sterilization, intrauterine device, injectable contraception, oral contraceptive pill, or male or female condom, emergency contraception, standard days method, or the lactational amenorrhea method. (14,15)

Our main outcome was unsuccessful implant discontinuation, measured by asking current implant users if they tried to have their current implant removed in the last 12 months (yes/no). Current implant users who reported trying to have their implant removed were categorized as experiencing an "unsuccessful" removal. Our secondary outcomes were reasons for and location of unsuccessful removal, which were ascertained by asking those with unsuccessful removal why they were not able to have their implant removed and where they went or who attempted to remove their implant. Responses to the latter were categorized as a public (i.e., government owned) or private-sector facility/provider.

Additionally, we estimated successful implant removal among women who were not currently using contraception and had used a method in the last 12 months. Current non-users reporting implant use within the last 12 months were classified, de facto, as having

"successful" implant removal[1]. Among women with a successful implant removal in the last 12 months, we also ascertained reasons for – and location of – successful implant removal, also delineating reliance on the public or private sector for this service. In the first three rounds of data included in this analysis (annually, 2016-2019), reason for removal was measured directly by asking women who reported implant removal in the last 12 months *"Why did you stop using your implant?"* For the most recent round of data collection in each country, this question was omitted, and both implant removal and reasons for removal were assessed using data from the contraceptive calendar (restricted to the 12 months preceding the interview). The contraceptive calendar is a retrospective instrument that documents duration of use and reasons for discontinuation, on a month-by-month basis, typically for multiple years.

Notably, not all measures related to implant removal were collected for all years in both countries. The location of successful removal was only captured for two years for each country (Burkina Faso: 2018 and 2019; Kenya: 2017 and 2018) and the location of unsuccessful removal was not collected in Burkina Faso in 2017 or Kenya in 2016. Additionally, reason for unsuccessful implant discontinuation was not collected in Kenya in 2017. Despite these limitations, we use all available data in each geography to examine trends and barriers to removal.

Analysis

We first describe sociodemographic characteristics of modern contraceptive users in each country for the most recent year of data. We use bivariate statistics and Pearson's chi-square tests to explore whether use of the implant versus any other modern method of contraception varied according to women's sociodemographic characteristics. To contextualize trends in access to removal, for each country and survey year, we describe the proportion of women currently using a contraceptive method, those using a modern method, and the overall contraceptive method mix.

Next, we estimate the proportion of current implant users who reported unsuccessful removal. We also estimate the proportion of all implant discontinuation attempts that were unsuccessful by dividing the total number of unsuccessful discontinuation attempts in the past 12 months by all discontinuation attempts in the past 12 months, both successful and unsuccessful. We use descriptive statistics to summarize locations of and reasons for successful and unsuccessful removal. Analyses were computed using Stata 16.0. Survey weights were applied to account for the probability of selection as well as non-response.

Ethical approval for PMA data collection activities was granted by in-country ethics committees in Burkina Faso (Comité d'Ethique pour la Recherche en Santé) and Kenya (Kenya Medical Research Institute (KEMRI) Ethics Review Committee). The Institutional Review Board for human subjects research at the Johns Hopkins Bloomberg School of Public Health reviewed and approved the study protocols for the PMA project.

[1] Due to limitations of the existing data (i.e., absence of a contraceptive calendar in select years in both countries), we are unable to estimate the proportion of successful implant removal among *currently* contracepting women (i.e., those women who removed an implant in the last 12 months and were using a different method at the time of data collection); we describe this limitation in further detail in the discussion section.

For peer review only

Results

Supplemental Table 1 provides background characteristics for all modern contraceptive users in the most recent year of data collection, stratified by use of implants versus other modern methods. Implants users, compared to other modern method users, were more likely to be older, married, less educated, live in rural areas and poorer households, have a greater number of children, and obtain their method from the public sector. While implant users in Kenya were more likely to prefer no more children compared to other modern method users (48% versus 44%, respectively), this difference was small, and no statistically significant differences were seen in the distribution of fertility preferences in Burkina Faso.

In Supplemental Table 2, we provide important context for trends in implant removal by presenting the prevalence of all contraception, modern contraception, and the method mix in each country for each year of data collection. The total number of participants ranged from 3,221 (2017) to 6,590 (2020) in Burkina Faso and from 5,864 (2017) to 9,469 (2019) in Kenya. In Burkina Faso, contraceptive prevalence increased in the first three years, from 23% in 2017 to 29% in 2019, while contraceptive prevalence remained stable at 45% in Kenya during this time.

Figures 1a-1b display trends in implant and injectable use. In Burkina Faso, implant use constituted 43% of the method mix in both 2017 (95% CI: 38%, 49%) and 2020 (95% CI: 39%, 48%) but the gap between implants and injectables (the second most common method) grew, as injectable use declined from 30% (95% CI: 23%, 39%) to 24% (95% CI: 19%, 31%). In Kenya, implant use grew from 28% (95% CI: 25%, 30%) in 2016 to 36% (95% CI: 34%, 38%) of the method mix in 2019. Due to a corresponding large decline in injectables (42% to 33%), implants are roughly tied with injectables as the primary contraceptive method in Kenya.

Figure 2a illustrates the percent of current implant users reporting an unsuccessful discontinuation attempt, and Figure 2b presents the percent of all implant discontinuation attempts that have been unsuccessful. In Burkina Faso, the percent of current implant users who reported an unsuccessful discontinuation attempt in the last 12 months declined from 7% (95% CI: 4%, 14%) in 2017 to 3% (95% CI: 2%, 6%) in 2020. In Kenya, the percent of current users reporting unsuccessful removal dropped from 9% (95% CI: 7%, 12%) in 2017 to 2% (95% CI: 1%, 3%) in 2019. Among all discontinuation attempts in Burkina Faso, the percent of removals that were unsuccessful dropped from 28% (95% CI: 19%, 40%) in 2017 to 15% (95% CI: 10%, 22%) in 2020. In contrast, Kenya dropped from 52% (95% CI: 44%, 61%) in 2017 to 13% (95% CI: 9%, 18%) in 2019.

Among women who had successfully removed their implant, we present reasons for removal in Burkina Faso (Figure 3a) and Kenya (Figure 3b). In Burkina Faso, reasons for removal varied over time, with women in 2016 most frequently reporting removal due to a desire to become pregnant (60%) or concerns about interference with the body's natural process (24%). Although these reasons were still commonly reported in later survey years, the proportion of women reporting these experiences diminished over time. By 2020, concerns over side effects were the primary reason for removal – reported by 42% of women - although more than one in three women still sought removal due to a desire to become pregnant. Method failure (became pregnant) was a frequent reason for removal in 2018 and 2019 but was reported by few women in 2017 and 2020. Very similar patterns were observed in Kenya, with a desire to become pregnant and health concerns/side effects consistently

reported by a large percent of respondents across all four rounds of data collection (29-49% and 27-48%, respectively), and up to half (42-51%) reporting method failure in 2017-2018.

Figure 4 presents the location of implant removal and discontinuation attempts, stratified by public versus private sector. Among Burkinabe women, removals were most frequently sourced from a public-sector facility for all years, for both successful removals (75-83%) and unsuccessful removals (85-95%). In Kenya, women sourced successful removal equally from both public and private sector locations in 2017 but far more commonly from the public-sector (79%) in 2018. Like Burkina Faso, most unsuccessful removals in Kenya were sourced from the public sector in all years (71-90%).

Among Burkinabe women who were unsuccessful in obtaining implant removal, self-reported barriers to removal fluctuated from year to year (Figure 5a). In 2017, Burkinabe women primarily reported that they were counseled against removal (23%), or a trained provider was unavailable (19%). The following year, women reported a provider refused to remove their implant (18%) or that they could not afford the cost of removal (28%). Cost remained a dominant barrier in 2019 but by 2020, nearly half of women (43%) reported they were counseled against removal, while more than one-third (36%) shared they were told to return to the facility another day. Similarly, in Kenya, common self-reported barriers shifted from year to year, with the highest proportion of women in 2016 and 2019 reporting a lack of a trained provider or cost, while in 2018 nearly half of women were counseled against removal or instructed to return another day (Figure 5b).

Discussion

In light of growing implant use and recent qualitative research documenting barriers to implant removal in sub-Saharan Africa, we explored changes over time in contraceptive use, implant use, and experiences with implant removal in Burkina Faso and Kenya. Over a four-year period, contraceptive prevalence was relatively steady in both settings. As a percentage of the overall method mix, implant use in Burkina Faso did not vary substantially from 2017 to 2020 (remaining around 43%) while injectable use occupied an *increasingly* distant second place. In contrast, Kenya experienced a steady increase in implant use, coupled with commensurate declines in injectable use.

Given the predominance of implants in both settings, access to method removal is imperative. We found that the percent of current implant users reporting an unsuccessful discontinuation attempt declined in both countries. Reasons for this trend are unclear. Given more than one out of every 10 women of reproductive age in each country is currently using a contraceptive implant, the 2% to 3% of current users unable to remove their implant translates into tens of thousands of women who experience structural forms of contraceptive coercion that prevent them from achieving their contraceptive goals.

In both Burkina Faso and Kenya, more than one in eight women seeking implant removal were unsuccessful in their attempt. In Kenya, this is evidence of a stark downward trend from one in two women just three years prior – suggesting considerable progress has been made to help reduce barriers to removal in Kenya; yet more progress is needed. The most common reasons women seek removal changed over time in both countries; however, wanting to get pregnant or becoming pregnant and concerns about the implant’s effect on the body and side effects were persistent reasons for desired removal in both countries. When women provided reasons for unsuccessful implant discontinuation, facility-level barriers were common,

For peer review only

including being counselled against removal, told a trained provider is unavailable, or asked to return another day. Cost was also a persistent barrier across time in both countries. Our results confirm prior findings that women encounter substantial facility-level barriers to implant removal as well as unaffordable removal fees, including within public facilities. (10,11,16–18)

Contraceptive implant users – compared to all other modern method users - were consistently older and more likely to be married, which may confer some degree of agency. However, they were also more likely to be uneducated, poor, and living in rural locations, where method removal may rely heavily on public-sector facilities. Women in both countries largely source implant removal – both successful and unsuccessful – from the public sector, where a nascent body of literature suggests providers are resistant to implant removal - at times to preserve limited implant supplies or sometimes due to an aversion for the procedure itself. Additionally, when noting the combination of cost as a substantial barrier and public facilities as the source, we posit a system of substantial informal fees for implant removal may enable providers to avoid removing implants, particularly among poor public-sector family planning clients who are especially price-sensitive. Informal payments are unofficial financial payments from patients to providers, over and above the official cost of services. A system of informal payments are well-documented in several countries in sub-Saharan Africa, including Kenya. (11,19)

This analysis benefits from multiple years of nationally representative data collected in diverse geographies. However, we acknowledge several limitations. First, we are limited by inconsistent survey items across all years of data. For example, in some years and for questions related to reasons for (or barriers to) implant removal, multiple responses were allowed, while in other years a single response option was used. This makes direct comparison of the frequency of reasons and barriers over time more difficult, as women may have multiple reasons for seeking or failing to have an implant removed but would have been unable to report all reasons in select survey years, resulting in under-reporting of secondary reasons. For example, we note a spike in reported method failure as a reason for discontinuation in two of the four years of data collection for each country and suspect the multiple response option provided in those years contributed to this inconsistency in reasons for removal, given the contraceptive implant has typically low failure rates. Similarly, the change in survey structure to include the contraceptive calendar in the most recent round of data collection resulted in minor differences in how women were asked about implant removal as well as under-reporting of implant discontinuation in the years preceding the calendar, during which only non-contraceptive users were asked about prior method use in the last 12 months. Second, small sample sizes for some of the analyses limited greater assessment of unsuccessful removal experiences. Third, among those with successful implant removal in the year prior to the survey, we were unable to measure whether these women first experienced a barrier to removal prior to successful removal. And, among those who were unsuccessful, we cannot report on the total number of unsuccessful attempts. This may have resulted in an underestimate of the percentage of current users that have experienced barriers to removal, presenting a conservative picture of the difficulty of implant removal. Further, these data do not capture implant removal experience of contraceptive switchers. Ideally, those women who switched from the implant to another method should be classified as having a successful removal, but current users were not asked about prior use in survey rounds without a calendar. As such, for select years we may have underestimated the number of successful removals, which would lead to an overestimation of the proportion of all removals that were unsuccessful. This is an important limitation of the currently available

For peer review only

data on implant removal and calls for improvements to existing survey instruments for measuring access to implant removal. We are additionally limited in this analysis by a lack of contextual information. For example, it is possible that an implant patient complaining of side effects may have received counseling to help mitigate side effects and may have continued implant use with satisfaction. We would not be able to capture this nuance with population-based data.

Despite these limitations, our findings are in line with those in other studies that have examined implant removal in sub-Saharan Africa. In a study following LARC users over one year, 5% of implant users in Nigeria and 7% of implant users in Zambia reported wanting their implants removed, but still using their method. (20) Another study in Ghana noted that only 55-61% of implant users who attempted removal successfully obtained removal on their first attempt. (21) These studies also found similar reasons for wanting implants removed, including side effects and desire for more children, and similar reasons for implant removal failure, including provider counselling to continue using the method. (20,21)

This analysis provides an important contribution to the literature on barriers to implant removal in sub-Saharan Africa. Using nationally representative data to assess changes over time in barriers to removal across two distinct countries, we note both much needed reductions in these barriers and also highlight the additional progress required to sufficiently safeguard contraceptive autonomy in LMICs. Our findings contribute to a growing body of literature highlighting the imperative for improved, patient-centered implant removal services in sub-Saharan Africa. (10,11,20,21) Despite international calls to ensure programmatic support for implant removal services scaled appropriately with the popularity of the implant, barriers to implant removal services persist. (2,9) Family planning programs and services must prioritize contraceptive autonomy, respecting a person's right to choose to discontinue their method at any time and for any reason.

Author Contributions

Data analysis was conceived by KT, LS, and CK, all of whom contributed to the analysis plan and paper framing. Quantitative analysis was conducted by BB and KT, with some input from LS and CK. KT, BB, SC, and EG all drafted portions of the initial manuscript. CK, LZ, PG, MT, GG, and the PMA PI Group contributed to data collection, data curation, data cleaning, and project administration. All authors contributed substantially to review and revision of the manuscript and have read and approved the final manuscript.

Declaration of interests

We declare no competing interest.

Data sharing

Access to the deidentified datasets used in this analysis are granted on a per request basis by Performance Monitoring and Action. To request datasets, please go to: https://www.pmadata.org/data/request-access-datasets

Ethical approval and consent to participate

Ethical approval for PMA data collection activities has been granted by in-country ethics committees in Burkina Faso (Comité d'Ethique pour la Recherche en Santé, #A14-2020) and Kenya (Kenya Medical Research Institute (KEMRI) Ethics Review Committee, #P241/04/2020). The Institutional Review Board for human subjects research at the Johns

For peer review only

Hopkins Bloomberg School of Public Health reviewed and approved the study protocols for the PMA project.

Funding and Acknowledgements

This work was supported, in part, by the Bill & Melinda Gates Foundation IVN 009639. Under the grant conditions of the Foundation, a Creative Commons Attribution 4.0 Generic License has already been assigned to the Author Accepted Manuscript version that might arise from this submission. Support for this research was also provided, in part, by a career development grant (R00 HD086270) to K. Tumlinson and an infrastructure grant for population research (P2C HD050924) to the Carolina Population Center at the University of North Carolina at Chapel Hill, both from The Eunice Kennedy Shriver National Institute of Child Health and Human Development (NICHD) of the National Institutes of Health (NIH). LS's contribution was supported by a Ruth L Kirschstein National Research Service Award (T32 HD049302) and Population Research Infrastructure grant (P2C HD047873) from the NICHD. BB's contribution was supported in part by funding from NICHD (T32 HD52468) and Population Infrastructure grant (P2C HD050924). Contributions by CK, LZ, PG, MT, GG, and the PMA PI Group were supported by grants OPP1198333 and OPP1198339 awarded by the Bill & Melinda Gates Foundation. We wish to gratefully acknowledge the support of Matt Gunther, research staff with IPUMS, for extracting and harmonizing several key variables from the PMA retrospective reproductive calendar data in Kenya and Burkina Faso. The contents of this article are solely the responsibility of the authors and do not necessarily represent the official views of the NIH/NICHD or the Bill & Melinda Gates Foundation.

The PMA project relies on the work of many individuals, both in the United States and in survey countries. The project team is grateful for support from the Bill & Melinda Gates Foundation and would like to thank the country teams and resident enumerators who are ultimately responsible for the success of PMA.

Figure 1a. Implants and injectables as a percent of the total modern contraceptive method mix, Burkina Faso

Figure 1b. Implants and injectables as a percent of the total modern contraceptive method mix, Kenya

Figure 2a. Percent of current implant users reporting unsuccessful discontinuation attempt

Figure 2b. Percent of all discontinuation attempts that were unsuccessful

Figure 3a. Reasons for implant discontinuation, Burkina Faso

Figure 3b. Reasons for implant discontinuation, Kenya

Figure 4. Location of implant discontinuation and discontinuation attempt in Burkina Faso and Kenya, 2017-2019

Figure 5a. Barriers to implant discontinuation, Burkina Faso

Figure 5b. Barriers to implant discontinuation, Kenya

For peer review only

References

1. Tumlinson K, Steiner MJ, Rademacher KH, Olawo A, Solomon M, Bratt J. The promise of affordable implants: is cost recovery possible in Kenya? Contraception. 2011 Jan;83(1):88–93.

2. Jacobstein R. Liftoff: the blossoming of contraceptive implant use in africa. Glob Health Sci Pract. 2018 Mar 21;6(1):17–39.

3. Winner B, Peipert JF, Zhao Q, Buckel C, Madden T, Allsworth JE, et al. Effectiveness of Long-Acting Reversible Contraception. Obstet Gynecol Surv. 2012 Sep;67(9):552–3.

4. Bahamondes L, Fernandes A, Monteiro I, Bahamondes MV. Long-acting reversible contraceptive (LARCs) methods. Best Pract Res Clin Obstet Gynaecol. 2020 Jul;66:28–40.

5. Kungu W, Khasakhala A, Agwanda A. Use of long-acting reversible contraception among adolescents and young women in Kenya. PLoS ONE. 2020 Nov 10;15(11):e0241506.

6. Kiemtoré S, Zoungrana Z, Zamané H, Kaboré CWPD, Ouédraogo A, Bonané B. Interventions to improve the use of long-acting reversible contraceptive methods at primary health centers in Burkina Faso. Int J Gynaecol Obstet. 2019 Dec;147(3):350–5.

7. Radovich E, Dennis ML, Barasa E, Cavallaro FL, Wong KL, Borghi J, et al. Who pays and how much? A cross-sectional study of out-of-pocket payment for modern contraception in Kenya. BMJ Open. 2019 Feb 20;9(2):e022414.

8. Ensuring human rights in the provision of contraceptive information and services: guidance and recommendations. Geneva: World Health Organization; 2014.

9. Christofield M, Lacoste M. Accessible Contraceptive Implant Removal Services: An Essential Element of Quality Service Delivery and Scale-Up. Glob Health Sci Pract. 2016 Sep 28;4(3):366–72.

10. Senderowicz L. “I was obligated to accept”: A qualitative exploration of contraceptive coercion. Soc Sci Med. 2019 Oct;239:112531.

11. Britton LE, Williams CR, Onyango D, Wambua D, Tumlinson K. “When it comes to time of removal, nothing is straightforward”: A qualitative study of experiences with barriers to removal of long-acting reversible contraception in Western Kenya. Contraception: X. 2021 Mar 22;3:100063.

12. Brunie A, Callahan RL, Mackenzie A, Kibira SPS, Wayack-Pambè M. Developing acceptable contraceptive methods: Mixed-method findings on preferred method

characteristics from Burkina Faso and Uganda. [version 2; peer review: 2 approved]. Gates Open Res. 2019 Apr 11;3:1205.

13. Zimmerman L, Olson H, PMA2020 Principal Investigators Group, Tsui A, Radloff S. PMA2020: Rapid Turn-Around Survey Data to Monitor Family Planning Service and Practice in Ten Countries. Stud Fam Plann. 2017 Aug 28;48(3):293–303.

14. World Health Organization Department of Reproductive Health and Research (WHO/RHR) and Johns Hopkins Bloomberg School of Public Health/Center for Communication Programs (CCP), Knowledge for Health Project. Family Planning: A Global Handbook for Providers (2018 Update). Updated 3rd. Baltimore and Geneva: CCP and WHO; 2021.

15. Festin MPR, Kiarie J, Solo J, Spieler J, Malarcher S, Van Look PFA, et al. Moving towards the goals of FP2020 - classifying contraceptives. Contraception. 2016 Oct;94(4):289–94.

16. Yirgu R, Wood SN, Karp C, Tsui A, Moreau C. "You better use the safer one… leave this one": the role of health providers in women's pursuit of their preferred family planning methods. BMC Womens Health. 2020 Aug 12;20(1):170.

17. Senderowicz L, Kolenda A. "She told me no, that you cannot change": Understanding provider refusal to remove contraceptive implants. SSM - Qualitative Research in Health. 2022 Dec;2:100154.

18. Senderowicz L, Karp C, Bullington BW, Tumlinson K, Zimmerman L, OlaOlorun FM, et al. Facility readiness to remove subdermal contraceptive implants in 6 sub-Saharan African countries. AJOG Glob Rep. 2022 Nov 5;2(4):100132.

19. Tumlinson K, Britton LE, Williams CR, Wambua DM, Otieno Onyango D. Informal payments for family planning: prevalence and perspectives of women, providers, and health sector key informants in western Kenya. Sexual and Reproductive Health Matters. 2021 Dec;29(1):1–17.

20. Brunie A, Stankevitz K, Nwala AA, Nqumayo M, Chen M, Danna K, et al. Expanding long-acting contraceptive options: a prospective cohort study of the hormonal intrauterine device, copper intrauterine device, and implants in Nigeria and Zambia. Lancet Glob Health. 2021 Oct;9(10):e1431–41.

21. Callahan R, Lebetkin E, Brennan C, Kuffour E, Boateng A, Tagoe S, et al. What Goes In Must Come Out: A Mixed-Method Study of Access to Contraceptive Implant Removal Services in Ghana. Glob Health Sci Pract. 2020 Jun 30;8(2):220–38.

For peer review only

Figure 1a: Implants and injectables as a percent of the total modern contraceptive method mix, Burkina Faso

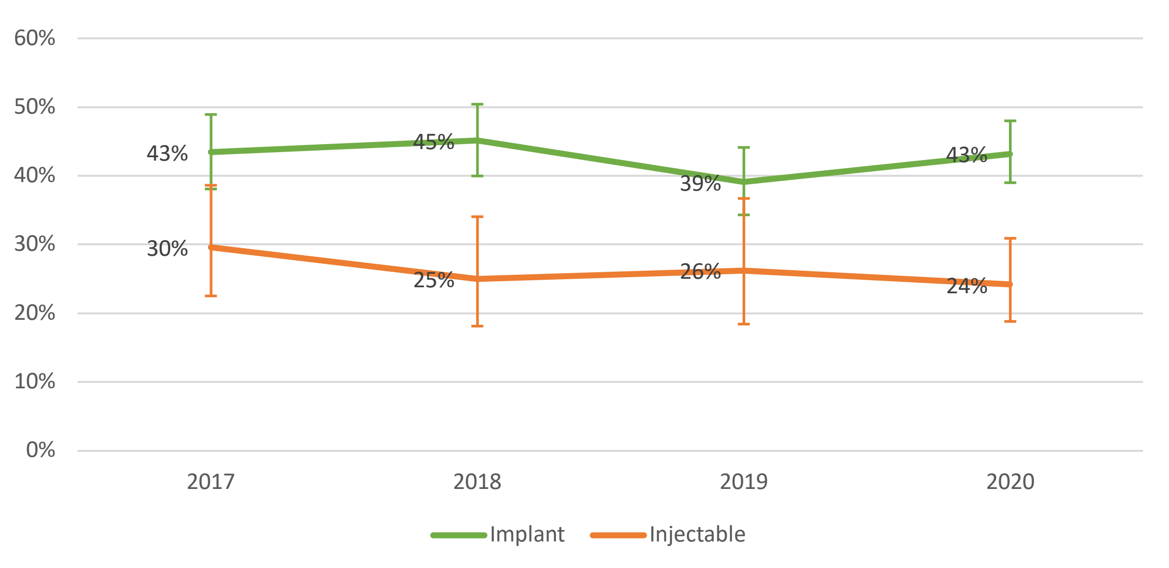

Figure 1b: Implants and injectables as a percent of the total modern contraceptive method mix, Kenya

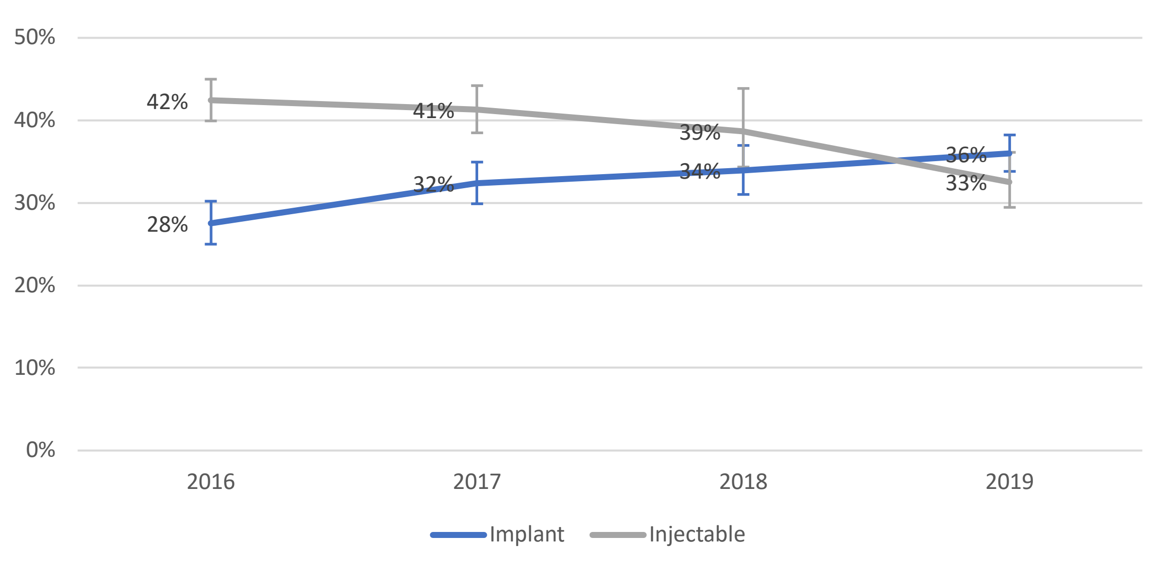

Figure 2a: Percent of current implant users reporting unsuccessful discontinuation attempt*

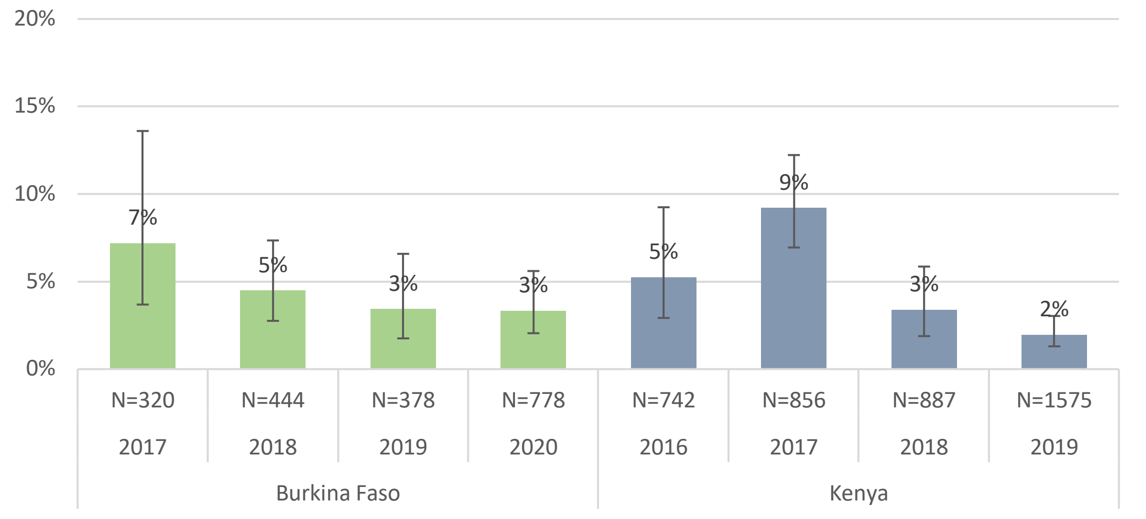

*Denominator is all current implant users.

Figure 2b: Percent of all discontinuation attempts that were unsuccessful*

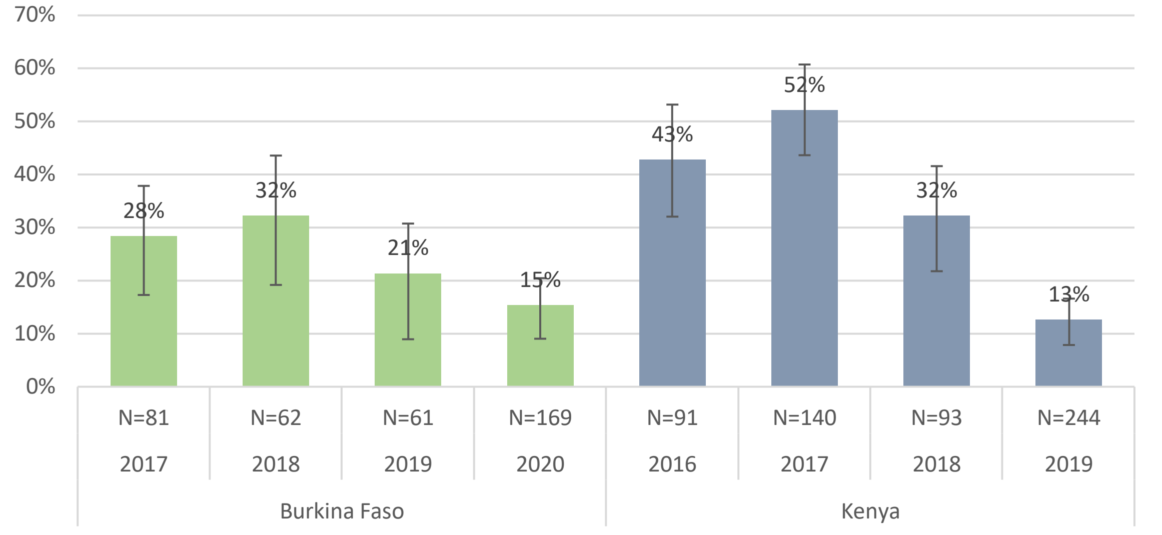

*Denominator is women with successful implant removal in the last 12 months and women with unsuccessful implant removal in the last 12 months.

Figure 3a: Reasons for implant discontinuation, Burkina Faso

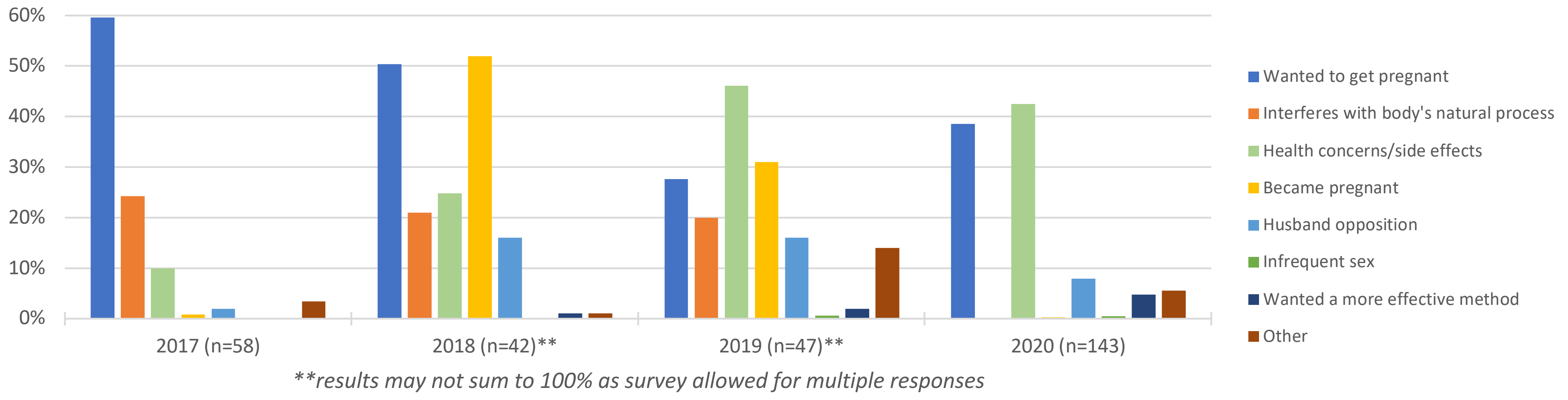

Figure 3b: Reasons for implant discontinuation, Kenya

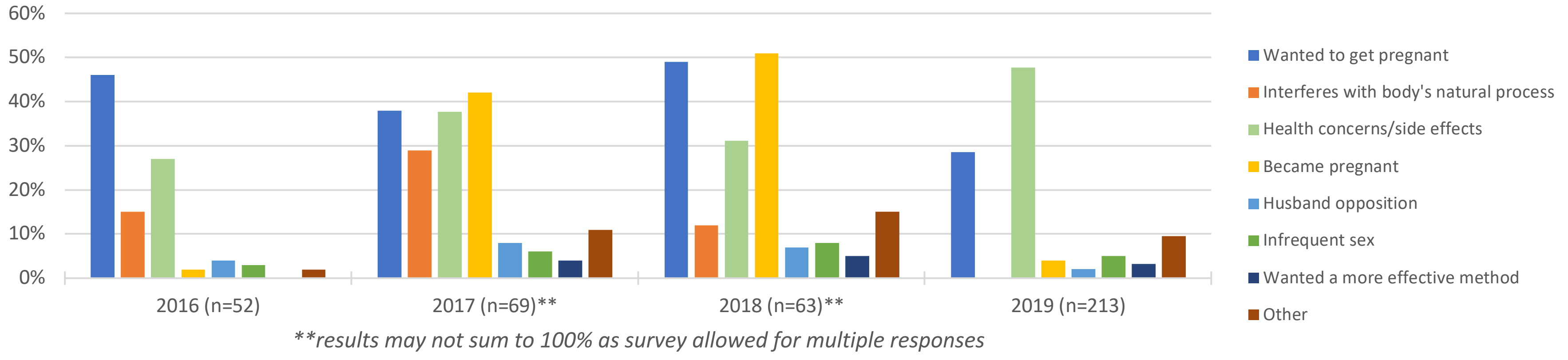

Figure 4: Location of implant discontinuation and discontinuation attempt in Burkina Faso and Kenya, 2017-2019

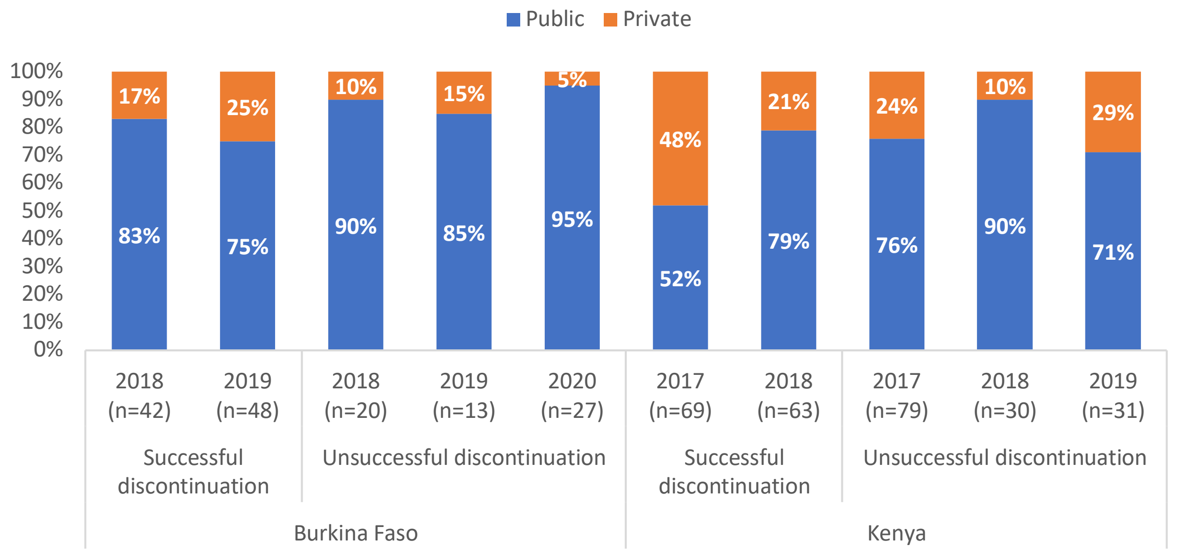

Figure 5a: Barriers to implant discontinuation, Burkina Faso

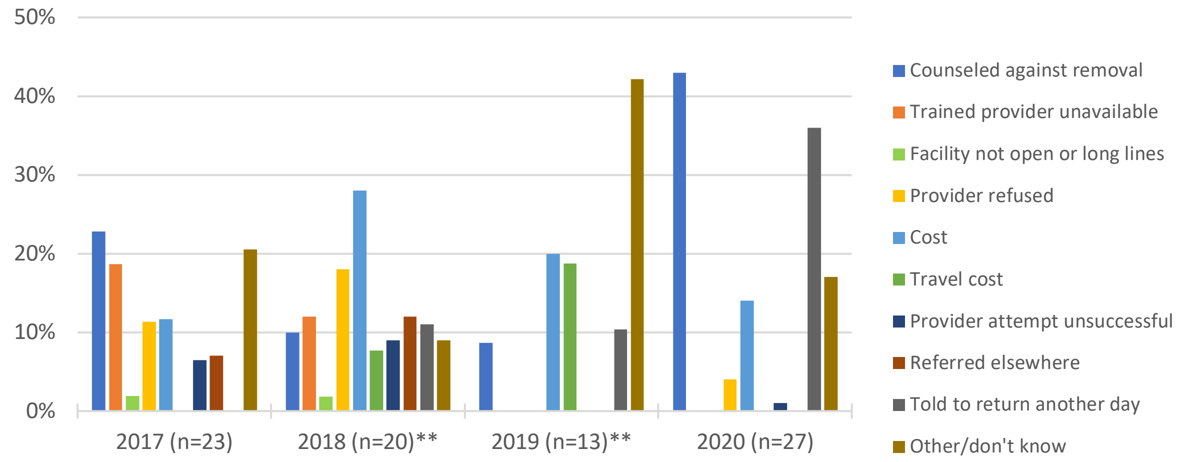

***results may not sum to 100% as survey allowed for multiple responses*

Figure 5b: Barriers to implant discontinuation, Kenya

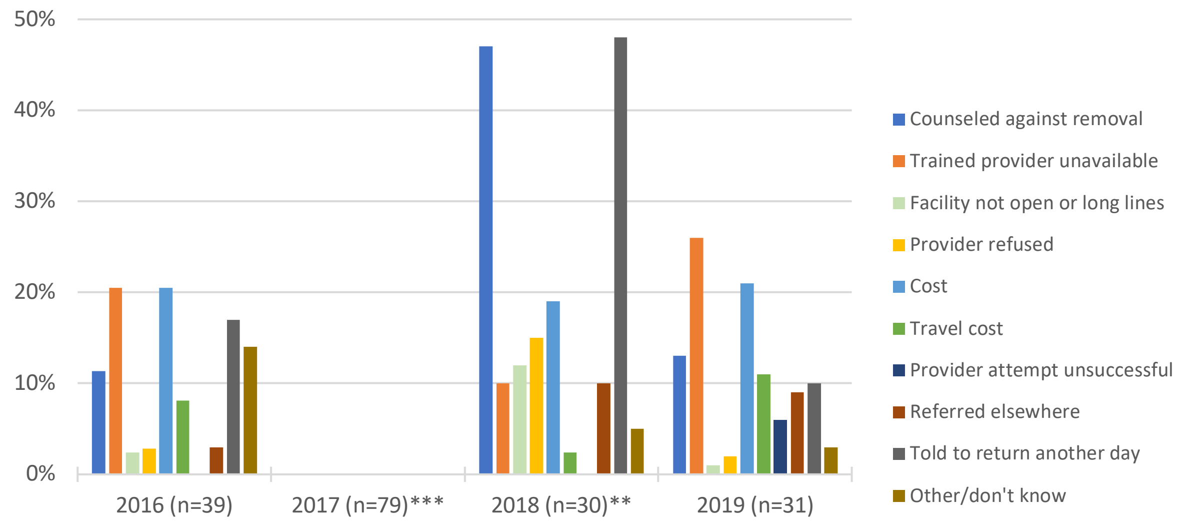

***results may not sum to 100% as survey allowed for multiple responses*
****question omitted*

Supplemental Table 1. Characteristics of modern method users compared to implant users, in Burkina Faso and Kenya. 2019.

	Burkina Faso			Kenya		
	2020			2019		
	All Other Modern	Implant	*p-value	All Other Modern	Implant	*p-value
**N**	900	778		2463	1575	
**Total % of all women**	14%	12%		26%	17%	
**Age**			0.300			<0.001
15-24	31%	29%		25%	25%	
25-39	55%	50%		55%	61%	
40-49	14%	22%		20%	14%	
**Marital status**			<0.001			0.001
In union	71%	82%		69%	78%	
Sexually active unmarried	29%	18%		31%	22%	
**Parity**			<0.001			<0.001
0	17%	4%		11%	1%	
1 to 2	29%	30%		37%	42%	
3 to 4	27%	25%		31%	34%	
5 or more	27%	41%		21%	23%	
**Residence**			<0.001			<0.001
Urban	37%	22%		36%	27%	
Rural	63%	78%		64%	73%	
**Education**			<0.001			<0.001
No education	46%	64%		2%	4%	
Primary	19%	19%		49%	57%	
Secondary	30%	16%		37%	30%	
Tertiary	4%	1%		13%	9%	
**Wealth tertile**			<0.001			<0.001
Lowest	25%	34%		28%	38%	
Middle	28%	34%		37%	36%	
Highest	47%	32%		35%	26%	
**Received from public facility**			<0.001			<0.001
Yes	74%	91%		61%	88%	
No	26%	9%		39%	12%	
**Fertility preferences**			0.090			<0.001
No more children	18%	25%		44%	48%	
Children in the next 2 years	35%	31%		16%	13%	
Children more than 2 years from now	38%	34%		33%	34%	
Says she can't get pregnant	1%	1%		2%	1%	
Undecided	8%	9%		5%	3%	
Other	1%	0%		0%	0%	

**We use the chi-square test to assess whether the distribution of characteristics differed based on whether a woman reported implant versus other modern method use.*

For peer review only

Supplemental Table 2. Contraceptive prevalence and method mix over time, among women of reproductive age in Burkina Faso and Kenya. 2016-2020

	Burkina Faso				Kenya			
	2017	2018	2019	2020	2016	2017	2018	2019
**N**	3221	3512	3329	6590	5891	5864	5658	9469
**Current contraceptive use**								
Yes	23%	28%	29%	27%	45%	45%	46%	45%
No	77%	72%	71%	73%	55%	55%	54%	55%
**Current modern contraceptive use**								
Yes	22%	26%	27%	25%	44%	44%	45%	43%
No	78%	74%	73%	75%	56%	56%	55%	57%
**N**	735	982	960	1800	2659	2592	2566	4271
**Method mix***								
Sterilization	0%	0%	0%	0%	4%	4%	4%	4%
IUD	3%	4%	4%	4%	5%	2%	4%	4%
Implant	43%	45%	39%	43%	28%	32%	34%	36%
Injectable	30%	25%	26%	24%	42%	41%	39%	33%
Pills	11%	11%	10%	9%	8%	8%	7%	7%
Condoms	8%	8%	13%	12%	6%	5%	5%	7%
Standard Days Method	0%	1%	0%	0%	0%	1%	0%	0%
Rhythm	3%	5%	5%	6%	2%	2%	2%	3%
Withdrawal	0%	0%	0%	1%	1%	1%	1%	1%
Emergency contraception	0%	0%	1%	0%	2%	3%	3%	2%
Other modern method	0%	0%	0%	1%	1%	0%	0%	1%
Other	1%	0%	1%	0%	0%	0%	0%	2%

*Among women reporting use of more than one method, only the most effective method is included in the calculation of the method mix.

For peer review only

STROBE 2007 (v4) Statement—Checklist of items that should be included in reports of *cross-sectional studies*

Section/Topic	Item #	Recommendation	Reported on page #
**Title and abstract**	1	(*a*) Indicate the study's design with a commonly used term in the title or the abstract	2
		(*b*) Provide in the abstract an informative and balanced summary of what was done and what was found	2
**Introduction**			
Background/rationale	2	Explain the scientific background and rationale for the investigation being reported	3
Objectives	3	State specific objectives, including any prespecified hypotheses	3
**Methods**			
Study design	4	Present key elements of study design early in the paper	4
Setting	5	Describe the setting, locations, and relevant dates, including periods of recruitment, exposure, follow-up, and data collection	4
Participants	6	(*a*) Give the eligibility criteria, and the sources and methods of selection of participants	4
Variables	7	Clearly define all outcomes, exposures, predictors, potential confounders, and effect modifiers. Give diagnostic criteria, if applicable	4-5
Data sources/ measurement	8*	For each variable of interest, give sources of data and details of methods of assessment (measurement). Describe comparability of assessment methods if there is more than one group	4-5
Bias	9	Describe any efforts to address potential sources of bias	*4-5*
Study size	10	Explain how the study size was arrived at	6
Quantitative variables	11	Explain how quantitative variables were handled in the analyses. If applicable, describe which groupings were chosen and why	4-5
Statistical methods	12	(*a*) Describe all statistical methods, including those used to control for confounding	5
		(*b*) Describe any methods used to examine subgroups and interactions	NA
		(*c*) Explain how missing data were addressed	4-5
		(*d*) If applicable, describe analytical methods taking account of sampling strategy	NA
		(*e*) Describe any sensitivity analyses	NA
**Results**			

For peer review only

Participants	13*	(a) Report numbers of individuals at each stage of study—eg numbers potentially eligible, examined for eligibility, confirmed eligible, included in the study, completing follow-up, and analysed	6
		(b) Give reasons for non-participation at each stage	6
		(c) Consider use of a flow diagram	NA
Descriptive data	14*	(a) Give characteristics of study participants (eg demographic, clinical, social) and information on exposures and potential confounders	6
		(b) Indicate number of participants with missing data for each variable of interest	6
Outcome data	15*	Report numbers of outcome events or summary measures	6-7
Main results	16	(*a*) Give unadjusted estimates and, if applicable, confounder-adjusted estimates and their precision (eg, 95% confidence interval). Make clear which confounders were adjusted for and why they were included	6-7
		(*b*) Report category boundaries when continuous variables were categorized	4-5
		(*c*) If relevant, consider translating estimates of relative risk into absolute risk for a meaningful time period	NA
Other analyses	17	Report other analyses done—eg analyses of subgroups and interactions, and sensitivity analyses	NA
**Discussion**			
Key results	18	Summarise key results with reference to study objectives	7
Limitations	19	Discuss limitations of the study, taking into account sources of potential bias or imprecision. Discuss both direction and magnitude of any potential bias	8
Interpretation	20	Give a cautious overall interpretation of results considering objectives, limitations, multiplicity of analyses, results from similar studies, and other relevant evidence	7-8
Generalisability	21	Discuss the generalisability (external validity) of the study results	7-8
**Other information**			
Funding	22	Give the source of funding and the role of the funders for the present study and, if applicable, for the original study on which the present article is based	9

*Give information separately for cases and controls in case-control studies and, if applicable, for exposed and unexposed groups in cohort and cross-sectional studies.

Note: An Explanation and Elaboration article discusses each checklist item and gives methodological background and published examples of transparent reporting. The STROBE checklist is best used in conjunction with this article (freely available on the Web sites of PLoS Medicine at http://www.plosmedicine.org/, Annals of Internal Medicine at http://www.annals.org/, and Epidemiology at http://www.epidem.com/). Information on the STROBE Initiative is available at www.strobe-statement.org.

For peer review only

BMJ Open

Assessing trends and reasons for unsuccessful implant discontinuation in Burkina Faso and Kenya between 2016 and 2020: A cross-sectional study

Journal:	*BMJ Open*
Manuscript ID	bmjopen-2023-071775.R2
Article Type:	Original research
Date Submitted by the Author:	26-Jun-2023

Complete List of Authors:	Tumlinson, Katherine; The University of North Carolina at Chapel Hill, Department of Maternal and Child Health<br>Senderowicz, Leigh ; University of Wisconsin-Madison, Department of Gender and Women's Studies<br>Bullington, Brooke; The University of North Carolina at Chapel Hill, Department of Epidemiology<br>Chung, Stephanie; The University of North Carolina at Chapel Hill, Department of Maternal and Child Health<br>Goland, Emilia; The University of North Carolina at Chapel Hill, Department of Maternal and Child Health<br>Zimmerman, Linnea; Johns Hopkins University Bloomberg School of Public Health, Department of Population, Family & Reproductive Health, Johns Hopkins Bloomberg School of Public Health, Baltimore, MD, USA<br>Gichangi, Peter; International Centre for Reproductive Health Kenya; University of Nairobi<br>Thiongo, Mary; International Centre for Reproductive Health Kenya<br>Guiella, Georges; ISSP/University of Ouagadougou)<br>NA, PMA Principal Investigators Group; NA<br>Karp, Celia; Johns Hopkins University Bloomberg School of Public Health

**Primary Subject Heading**:	Obstetrics and gynaecology
Secondary Subject Heading:	Public health, Reproductive medicine
Keywords:	REPRODUCTIVE MEDICINE, PUBLIC HEALTH, EPIDEMIOLOGY, Quality in health care < HEALTH SERVICES ADMINISTRATION & MANAGEMENT

SCHOLARONE™
Manuscripts

I, the Submitting Author has the right to grant and does grant on behalf of all authors of the Work (as defined in the below author licence), an exclusive licence and/or a non-exclusive licence for contributions from authors who are: i) UK Crown employees; ii) where BMJ has agreed a CC-BY licence shall apply, and/or iii) in accordance with the terms applicable for US Federal Government officers or employees acting as part of their official duties; on a worldwide, perpetual, irrevocable, royalty-free basis to BMJ Publishing Group Ltd ("BMJ") its licensees and where the relevant Journal is co-owned by BMJ to the co-owners of the Journal, to publish the Work in this journal and any other BMJ products and to exploit all rights, as set out in our licence.

The Submitting Author accepts and understands that any supply made under these terms is made by BMJ to the Submitting Author unless you are acting as an employee on behalf of your employer or a postgraduate student of an affiliated institution which is paying any applicable article publishing charge ("APC") for Open Access articles. Where the Submitting Author wishes to make the Work available on an Open Access basis (and intends to pay the relevant APC), the terms of reuse of such Open Access shall be governed by a Creative Commons licence – details of these licences and which Creative Commons licence will apply to this Work are set out in our licence referred to above.

Other than as permitted in any relevant BMJ Author's Self Archiving Policies, I confirm this Work has not been accepted for publication elsewhere, is not being considered for publication elsewhere and does not duplicate material already published. I confirm all authors consent to publication of this Work and authorise the granting of this licence.

For peer review only

Title: Assessing trends and reasons for unsuccessful implant discontinuation in Burkina Faso and Kenya between 2016 and 2020: A cross-sectional study

Authors: Katherine Tumlinson[1,2], Leigh Senderowicz[3], Brooke W. Bullington[2,4], Stephanie Chung[1], Emilia Goland[1], Linnea Zimmerman[5], Peter Gichangi[6], Mary Thiongo[6], Georges Guiella[7], PMA Principal Investigators Group[#], Celia Karp[5]

Affiliations:
[1] Department of Maternal and Child Health, Gillings School of Global Public Health, University of North Carolina at Chapel Hill
[2] Carolina Population Center, University of North Carolina at Chapel Hill
[3] Department of Obstetrics and Gynecology, University of Wisconsin-Madison
[4] Department of Epidemiology, Gillings School of Global Public Health, University of North Carolina at Chapel Hill
[5] Department of Population Family and Reproductive Health, Johns Hopkins Bloomberg School of Public Health, Baltimore, Maryland, USA.
[6] International Centre for Reproductive Health-Kenya, Nairobi, Kenya
[7] Institut Supérieur des Sciences de la Population (ISSP/University of Ouagadougou), Ouagadougou, Burkina Faso
The following members of the Performance Monitoring for Action (PMA) Principal Investigators group contributed to the data collection and development of this manuscript: Solomon Shiferaw and Assefa Seme, Addis Ababa University, Ethiopia; Elizabeth Omoluabi, Statistics and Population Studies Department, University of the Western Cape, South Africa; Fredrick Edward Makumbi and Simon Kibera, Makerere University School of Public Health, Uganda.

Corresponding Author:
Brooke W. Bullington
Department of Epidemiology
Gillings School of Global Public Health
University of North Carolina at Chapel Hill
bbullington@unc.edu

Key words: Contraceptive coercion, Contraceptive implants, Implant removal, Reproductive justice, long-acting reversible contraception (LARC), sub-Saharan Africa

Word count: 3,493

For peer review only

ABSTRACT

Objectives

Contraceptive implant use has grown considerably in the last decade, particularly among women in Burkina Faso and Kenya, where implant use is among the highest globally. We aim to quantify the proportion of current implant users who have unsuccessfully attempted implant removal in Burkina Faso and Kenya and document reasons for and location of unsuccessful removal.

Methods

We use nationally representative data collected between 2016 and 2020 from a cross-section of women of reproductive age in Burkina Faso and Kenya to estimate the prevalence of implant use, proportion of current implant users who unsuccessfully attempted removal, and proportion of all removal attempts that have been unsuccessful. We describe reasons for and barriers to removal, including the type of facility where successful and unsuccessful attempts occurred.

Findings

The total number of participants ranged from 3,221 (2017) to 6,590 (2020) in Burkina Faso and from 5,864 (2017) to 9,469 (2019) in Kenya. Over a four-year period, the percentage of current implant users reporting an unsuccessful implant discontinuation declined from 9% (95% CI: 7%, 12%) to 2% (95% CI: 1%, 3%) in Kenya and from 7% (95% CI: 4%, 14%) to 3% (95% CI: 2%, 6%) in Burkina Faso. Common barriers to removal included being counseled against removal by the provider or told to return a different day.

Conclusion

Unsuccessful implant discontinuation has decreased in recent years. Despite progress, substantial numbers of women desire having their contraceptive implant removed but are unable to do so. Greater attention to health systems barriers preventing implant removal is imperative to protecting reproductive autonomy and ensuring women can achieve their reproductive goals.

STRENGTHS AND LIMITATIONS

- This analysis uses nationally representative Performance Monitoring for Action data from multiple years across diverse geographies.
- Survey items were asked inconsistently between 2016 and 2020, making direct comparisons difficult in some instances. For example, comparison of reasons and barriers for implant removal was difficult to compare over time.
- Small sample sizes of women seeking implant removal limited greater assessment of unsuccessful removal experiences.
- Data do not capture implant removal experiences of women who switched contraceptive methods.

Background

Access to contraceptive implants has increased substantially in many low- and middle-income countries (LMICs) over the past decade. Political and financial commitments to contraceptive access from governments and international donors has led to increased supply and reduced commodity cost of implants. This has resulted in expanded access to implants in LMICs, diversifying the contraceptive method mix and contributing to substantial growth in the contraceptive prevalence rate, particularly in sub-Saharan Africa. (1,2) Implants, a form of long-acting reversible contraception (LARC), are highly effective, easy to maintain, and relatively low cost over time, making them a popular choice for many women in sub-Saharan Africa. (3,4) In Burkina Faso and Kenya, patterns in contraceptive use have changed dramatically in recent years, as many women have shifted from using injectable contraception to implants. (2,5) In both countries, implants are heavily subsidized by the government and donors, and are provided free of charge or at a low cost. (6,7)

While contraceptive implants offer many advantages, insertion and removal require a trained clinician, posing potential barriers to successful uptake and discontinuation. To uphold principles of human rights, provision of reliable, sustainable, and equitable services for both insertion and removal of implants must be ensured. Lack of access to high-quality implant removal services compromises reproductive autonomy, particularly for individuals who wish to stop using the method. (8) With increased uptake of implants, the demand for removal service in sub-Saharan Africa is estimated to increase substantially in the coming years, yet the extent to which individuals can access such services, as well as barriers they may face in seeking removal, has not been readily researched. (9) Additionally, understanding the unique sociodemographic characteristics of implant users can provide important insights into the potential implications of such barriers.

A 2019 qualitative study shed light on several barriers to person-centered care, specifically for individuals receiving LARC methods. Women from a sub-Saharan African country described being pressured to adopt LARC they did not want or fully understand due to limited choices, biased counselling, scare tactics, and misinformation from service providers. Several women reported being denied removal services before completing the five-year contraceptive efficacy period for the method, despite their desires for removal, even if they wished to become pregnant. (10) Similar findings emerged in a qualitative study from Western Kenya, where women shared experiences of being refused removal and pressured to use LARC for its full duration, regardless of their reported side effects. Other barriers to removal services included high removal costs, both in terms of formal and informal fees. (11) In a mixed-method study among women in Burkina Faso and Uganda, respondents reported anxiety over the potential added costs of method removal. (12) While qualitative evidence demonstrates barriers women face when seeking removal, few quantitative studies have estimated this experience among population-based samples of current and recent implant users, limiting our understanding of the frequency and reasons for unsuccessful removals.

This study seeks to expand upon recent qualitative findings highlighting substantial barriers to implant removal. Using nationally representative data collected between 2016 and 2020 in Burkina Faso and Kenya, we examine sociodemographic characteristics of implant users, recent trends in implant use, unsuccessful removal, and barriers to successful removal. We aim to quantify the proportion of current implant users who have unsuccessfully attempted implant removal in each country, and document reasons for and location of unsuccessful removal over the four-year period.

For peer review only

Methods

Data Source and Sample

This cross-sectional study uses multiple rounds of survey data from the Performance Monitoring for Action (PMA) project, formerly known as PMA2020. Since 2019, PMA has conducted yearly population-based surveys with women of reproductive age (15-49 years), in 10 countries across sub-Saharan Africa and South Asia, generating data on key reproductive health indicators. Data from PMA2020 on implant removal are available in select countries dating back to 2016. We restrict our analysis to the two countries that measured implant removal experiences across multiple rounds of data from PMA2020 and PMA, and for which implants constitute the most widely used method. Based on these criteria, we include data collected in Burkina Faso and Kenya between 2016 and 2020, with each country contributing four rounds of data. Participating women were selected using a multistage sampling strategy. All eligible women were invited to participate via an informed consent process. Enumerators collected data from each participant, including background characteristics, reproductive and contraceptive behaviors, and experiences with contraceptive health services (e.g., implant removal). Additional details of PMA's survey methodology have been published elsewhere and can be found at https://www.pmadata.org/data/survey-methodology. (13)

Patient and Public Involvement

Patients and the public were not involved in the design, conduct, reporting, or dissemination plans of our research.

Measures

Our primary measures assess women's contraceptive use and, among users of the contraceptive implant, experiences of removal. To assess changes in contraceptive use, implant use, and the contraceptive method mix since 2016, we used women's responses to the question, "*Are you or your partner currently doing something or using any method to delay or avoid getting pregnant?*" Among women who reported use, they were asked to specify which method they were currently using. We compare use of the implant to use of other modern methods, defined as female or male sterilization, intrauterine device, injectable contraception, oral contraceptive pill, or male or female condom, emergency contraception, standard days method, or the lactational amenorrhea method. (14,15)

Our main outcome was unsuccessful implant discontinuation, measured by asking current implant users if they tried to have their current implant removed in the last 12 months (yes/no). Current implant users who reported trying to have their implant removed were categorized as experiencing an "unsuccessful" removal. Our secondary outcomes were reasons for and location of unsuccessful removal, which were ascertained by asking those with unsuccessful removal why they were not able to have their implant removed and where they went or who attempted to remove their implant. Responses to the latter were categorized as a public (i.e., government owned) or private-sector facility/provider.

Additionally, we estimated successful implant removal among women who were not currently using contraception and had used a method in the last 12 months. Current non-users reporting implant use within the last 12 months were classified, de facto, as having

"successful" implant removal[1]. Among women with a successful implant removal in the last 12 months, we also ascertained reasons for – and location of – successful implant removal, also delineating reliance on the public or private sector for this service. In the first three rounds of data included in this analysis (annually, 2016-2019), reason for removal was measured directly by asking women who reported implant removal in the last 12 months *"Why did you stop using your implant?"* For the most recent round of data collection in each country, this question was omitted, and both implant removal and reasons for removal were assessed using data from the contraceptive calendar (restricted to the 12 months preceding the interview). The contraceptive calendar is a retrospective instrument that documents duration of use and reasons for discontinuation, on a month-by-month basis, typically for multiple years.

Notably, not all measures related to implant removal were collected for all years in both countries. The location of successful removal was only captured for two years for each country (Burkina Faso: 2018 and 2019; Kenya: 2017 and 2018) and the location of unsuccessful removal was not collected in Burkina Faso in 2017 or Kenya in 2016. Additionally, reason for unsuccessful implant discontinuation was not collected in Kenya in 2017. Despite these limitations, we use all available data in each geography to examine trends and barriers to removal.

Analysis

We first describe sociodemographic characteristics of modern contraceptive users in each country for the most recent year of data. We use bivariate statistics and Pearson's chi-square tests to explore whether use of the implant versus any other modern method of contraception varied according to women's sociodemographic characteristics. To contextualize trends in access to removal, for each country and survey year, we describe the proportion of women currently using a contraceptive method, those using a modern method, and the overall contraceptive method mix.

Next, we estimate the proportion of current implant users who reported unsuccessful removal. We also estimate the proportion of all implant discontinuation attempts that were unsuccessful by dividing the total number of unsuccessful discontinuation attempts in the past 12 months by all discontinuation attempts in the past 12 months, both successful and unsuccessful. We use descriptive statistics to summarize locations of and reasons for successful and unsuccessful removal. Analyses were computed using Stata 16.0. Survey weights were applied to account for the probability of selection as well as non-response.

Ethical approval for PMA data collection activities was granted by in-country ethics committees in Burkina Faso (Comité d'Ethique pour la Recherche en Santé) and Kenya (Kenya Medical Research Institute (KEMRI) Ethics Review Committee). The Institutional Review Board for human subjects research at the Johns Hopkins Bloomberg School of Public Health reviewed and approved the study protocols for the PMA project.

---

[1] Due to limitations of the existing data (i.e., absence of a contraceptive calendar in select years in both countries), we are unable to estimate the proportion of successful implant removal among *currently* contracepting women (i.e., those women who removed an implant in the last 12 months and were using a different method at the time of data collection); we describe this limitation in further detail in the discussion section.

For peer review only

Results

Supplemental Table 1 provides background characteristics for all modern contraceptive users in the most recent year of data collection, stratified by use of implants versus other modern methods. Implants users, compared to other modern method users, were more likely to be older, married, less educated, live in rural areas and poorer households, have a greater number of children, and obtain their method from the public sector. While implant users in Kenya were more likely to prefer no more children compared to other modern method users (48% versus 44%, respectively), this difference was small, and no statistically significant differences were seen in the distribution of fertility preferences in Burkina Faso.

In Supplemental Table 2, we provide important context for trends in implant removal by presenting the prevalence of all contraception, modern contraception, and the method mix in each country for each year of data collection. The total number of participants ranged from 3,221 (2017) to 6,590 (2020) in Burkina Faso and from 5,864 (2017) to 9,469 (2019) in Kenya. In Burkina Faso, contraceptive prevalence increased in the first three years, from 23% in 2017 to 29% in 2019, while contraceptive prevalence remained stable at 45% in Kenya during this time.

Figures 1a-1b display trends in implant and injectable use. In Burkina Faso, implant use constituted 43% of the method mix in both 2017 (95% CI: 38%, 49%) and 2020 (95% CI: 39%, 48%) but the gap between implants and injectables (the second most common method) grew, as injectable use declined from 30% (95% CI: 23%, 39%) to 24% (95% CI: 19%, 31%). In Kenya, implant use grew from 28% (95% CI: 25%, 30%) in 2016 to 36% (95% CI: 34%, 38%) of the method mix in 2019. Due to a corresponding large decline in injectables (42% to 33%), implants are roughly tied with injectables as the primary contraceptive method in Kenya.

Figure 2a illustrates the percent of current implant users reporting an unsuccessful discontinuation attempt, and Figure 2b presents the percent of all implant discontinuation attempts that have been unsuccessful. In Burkina Faso, the percent of current implant users who reported an unsuccessful discontinuation attempt in the last 12 months declined from 7% (95% CI: 4%, 14%) in 2017 to 3% (95% CI: 2%, 6%) in 2020. In Kenya, the percent of current users reporting unsuccessful removal dropped from 9% (95% CI: 7%, 12%) in 2017 to 2% (95% CI: 1%, 3%) in 2019. Among all discontinuation attempts in Burkina Faso, the percent of removals that were unsuccessful dropped from 28% (95% CI: 19%, 40%) in 2017 to 15% (95% CI: 10%, 22%) in 2020. In contrast, Kenya dropped from 52% (95% CI: 44%, 61%) in 2017 to 13% (95% CI: 9%, 18%) in 2019.

Among women who had successfully removed their implant, we present reasons for removal in Burkina Faso (Figure 3a) and Kenya (Figure 3b). In Burkina Faso, reasons for removal varied over time, with women in 2016 most frequently reporting removal due to a desire to become pregnant (60%) or concerns about interference with the body's natural process (24%). Although these reasons were still commonly reported in later survey years, the proportion of women reporting these experiences diminished over time. By 2020, concerns over side effects were the primary reason for removal – reported by 42% of women - although more than one in three women still sought removal due to a desire to become pregnant. Method failure (became pregnant) was a frequent reason for removal in 2018 and 2019 but was reported by few women in 2017 and 2020. Very similar patterns were observed in Kenya, with a desire to become pregnant and health concerns/side effects consistently

reported by a large percent of respondents across all four rounds of data collection (29-49% and 27-48%, respectively), and up to half (42-51%) reporting method failure in 2017-2018.

Figure 4 presents the location of implant removal and discontinuation attempts, stratified by public versus private sector. Among Burkinabe women, removals were most frequently sourced from a public-sector facility for all years, for both successful removals (75-83%) and unsuccessful removals (85-95%). In Kenya, women sourced successful removal equally from both public and private sector locations in 2017 but far more commonly from the public-sector (79%) in 2018. Like Burkina Faso, most unsuccessful removals in Kenya were sourced from the public sector in all years (71-90%).

Among Burkinabe women who were unsuccessful in obtaining implant removal, self-reported barriers to removal fluctuated from year to year (Figure 5a). In 2017, Burkinabe women primarily reported that they were counseled against removal (23%), or a trained provider was unavailable (19%). The following year, women reported a provider refused to remove their implant (18%) or that they could not afford the cost of removal (28%). Cost remained a dominant barrier in 2019 but by 2020, nearly half of women (43%) reported they were counseled against removal, while more than one-third (36%) shared they were told to return to the facility another day. Similarly, in Kenya, common self-reported barriers shifted from year to year, with the highest proportion of women in 2016 and 2019 reporting a lack of a trained provider or cost, while in 2018 nearly half of women were counseled against removal or instructed to return another day (Figure 5b).

Discussion

In light of growing implant use and recent qualitative research documenting barriers to implant removal in sub-Saharan Africa, we explored changes over time in contraceptive use, implant use, and experiences with implant removal in Burkina Faso and Kenya. Over a four-year period, contraceptive prevalence was relatively steady in both settings. As a percentage of the overall method mix, implant use in Burkina Faso did not vary substantially from 2017 to 2020 (remaining around 43%) while injectable use occupied an *increasingly* distant second place. In contrast, Kenya experienced a steady increase in implant use, coupled with commensurate declines in injectable use.

Given the predominance of implants in both settings, access to method removal is imperative. We found that the percent of current implant users reporting an unsuccessful discontinuation attempt declined in both countries. Reasons for this trend are unclear. Given more than one out of every 10 women of reproductive age in each country is currently using a contraceptive implant, the 2% to 3% of current users unable to remove their implant translates into tens of thousands of women who experience structural forms of contraceptive coercion that prevent them from achieving their contraceptive goals.

In both Burkina Faso and Kenya, more than one in eight women seeking implant removal were unsuccessful in their attempt. In Kenya, this is evidence of a stark downward trend from one in two women just three years prior – suggesting considerable progress has been made to help reduce barriers to removal in Kenya; yet more progress is needed. The most common reasons women seek removal changed over time in both countries; however, wanting to get pregnant or becoming pregnant and concerns about the implant's effect on the body and side effects were persistent reasons for desired removal in both countries. When women provided reasons for unsuccessful implant discontinuation, facility-level barriers were common,

For peer review only

including being counselled against removal, told a trained provider is unavailable, or asked to return another day. Cost was also a persistent barrier across time in both countries. Our results confirm prior findings that women encounter substantial facility-level barriers to implant removal as well as unaffordable removal fees, including within public facilities. (10,11,16–18)

Contraceptive implant users – compared to all other modern method users - were consistently older and more likely to be married, which may confer some degree of agency. However, they were also more likely to be uneducated, poor, and living in rural locations, where method removal may rely heavily on public-sector facilities. Women in both countries largely source implant removal – both successful and unsuccessful – from the public sector, where a nascent body of literature suggests providers are resistant to implant removal - at times to preserve limited implant supplies or sometimes due to an aversion for the procedure itself. Additionally, when noting the combination of cost as a substantial barrier and public facilities as the source, we posit a system of substantial informal fees for implant removal may enable providers to avoid removing implants, particularly among poor public-sector family planning clients who are especially price-sensitive. Informal payments are unofficial financial payments from patients to providers, over and above the official cost of services. Systems of informal payments are well-documented in several countries in sub-Saharan Africa, including Kenya. (11,19)

This analysis benefits from multiple years of nationally representative data collected in diverse geographies. However, we acknowledge several limitations. First, we are limited by inconsistent survey items across all years of data. For example, in some years and for questions related to reasons for (or barriers to) implant removal, multiple responses were allowed, while in other years a single response option was used. This makes direct comparison of the frequency of reasons and barriers over time more difficult, as women may have multiple reasons for seeking or failing to have an implant removed but would have been unable to report all reasons in select survey years, resulting in under-reporting of secondary reasons. For example, we note a spike in reported method failure as a reason for discontinuation in two of the four years of data collection for each country and suspect the multiple response option provided in those years contributed to this inconsistency in reasons for removal, given the contraceptive implant has typically low failure rates. Similarly, the change in survey structure to include the contraceptive calendar in the most recent round of data collection resulted in minor differences in how women were asked about implant removal as well as under-reporting of implant discontinuation in the years preceding the calendar, during which only non-contraceptive users were asked about prior method use in the last 12 months. Second, small sample sizes for some of the analyses limited greater assessment of unsuccessful removal experiences. Third, among those with successful implant removal in the year prior to the survey, we were unable to measure whether these women first experienced a barrier to removal prior to successful removal. And, among those who were unsuccessful, we cannot report on the total number of unsuccessful attempts. This may have resulted in an underestimate of the percentage of current users that have experienced barriers to removal, presenting a conservative picture of the difficulty of implant removal. Further, these data do not capture implant removal experience of contraceptive switchers. Ideally, those women who switched from the implant to another method should be classified as having a successful removal, but current users were not asked about prior use in survey rounds without a calendar. As such, for select years we may have underestimated the number of successful removals, which would lead to an overestimation of the proportion of all removals that were unsuccessful. This is an important limitation of the currently available

data on implant removal and calls for improvements to existing survey instruments for measuring access to implant removal. We are additionally limited in this analysis by a lack of contextual information. For example, it is possible that an implant patient complaining of side effects may have received counseling to help mitigate side effects and may have continued implant use with satisfaction. We would not be able to capture this nuance with population-based data.

Despite these limitations, our findings are in line with those in other studies that have examined implant removal in sub-Saharan Africa. In a study following LARC users over one year, 5% of implant users in Nigeria and 7% of implant users in Zambia reported wanting their implants removed, but still using their method. (20) Another study in Ghana noted that only 55-61% of implant users who attempted removal successfully obtained removal on their first attempt. (21) These studies also found similar reasons for wanting implants removed, including side effects and desire for more children, and similar reasons for implant removal failure, including provider counselling to continue using the method. (20,21)

This analysis provides an important contribution to the literature on barriers to implant removal in sub-Saharan Africa. Using nationally representative data to assess changes over time in barriers to removal across two distinct countries, we note both much needed reductions in these barriers and also highlight the additional progress required to sufficiently safeguard contraceptive autonomy in LMICs. Our findings contribute to a growing body of literature highlighting the imperative for improved, patient-centered implant removal services in sub-Saharan Africa. (10,11,20,21) Despite international calls to ensure programmatic support for implant removal services scaled appropriately with the popularity of the implant, barriers to implant removal services persist. (2,9) Family planning programs and services must prioritize contraceptive autonomy, respecting a person's right to choose to discontinue their method at any time and for any reason.

Author Contributions

Data analysis was conceived by KT, LS, and CK, all of whom contributed to the analysis plan and paper framing. Quantitative analysis was conducted by BB and KT, with some input from LS and CK. KT, BB, SC, and EG all drafted portions of the initial manuscript. CK, LZ, PG, MT, GG, and the PMA PI Group contributed to data collection, data curation, data cleaning, and project administration. All authors contributed substantially to review and revision of the manuscript and have read and approved the final manuscript.

Declaration of interests

We declare no competing interest.

Data sharing

Access to the deidentified datasets used in this analysis are granted on a per request basis by Performance Monitoring and Action. To request datasets, please go to: https://www.pmadata.org/data/request-access-datasets

Ethical approval and consent to participate

Ethical approval for PMA data collection activities has been granted by in-country ethics committees in Burkina Faso (Comité d'Ethique pour la Recherche en Santé, #A14-2020) and Kenya (Kenya Medical Research Institute (KEMRI) Ethics Review Committee, #P241/04/2020). The Institutional Review Board for human subjects research at the Johns

For peer review only

Hopkins Bloomberg School of Public Health reviewed and approved the study protocols for the PMA project.

Funding and Acknowledgements
This work was supported, in part, by the Bill & Melinda Gates Foundation IVN 009639. Under the grant conditions of the Foundation, a Creative Commons Attribution 4.0 Generic License has already been assigned to the Author Accepted Manuscript version that might arise from this submission. Support for this research was also provided, in part, by a career development grant (R00 HD086270) to K. Tumlinson and an infrastructure grant for population research (P2C HD050924) to the Carolina Population Center at the University of North Carolina at Chapel Hill, both from The Eunice Kennedy Shriver National Institute of Child Health and Human Development (NICHD) of the National Institutes of Health (NIH). LS's contribution was supported by a Ruth L Kirschstein National Research Service Award (T32 HD049302) and Population Research Infrastructure grant (P2C HD047873) from the NICHD. BB's contribution was supported in part by funding from NICHD (T32 HD52468) and Population Infrastructure grant (P2C HD050924). Contributions by CK, LZ, PG, MT, GG, and the PMA PI Group were supported by grants OPP1198333 and OPP1198339 awarded by the Bill & Melinda Gates Foundation. We wish to gratefully acknowledge the support of Matt Gunther, research staff with IPUMS, for extracting and harmonizing several key variables from the PMA retrospective reproductive calendar data in Kenya and Burkina Faso. The contents of this article are solely the responsibility of the authors and do not necessarily represent the official views of the NIH/NICHD or the Bill & Melinda Gates Foundation.

The PMA project relies on the work of many individuals, both in the United States and in survey countries. The project team is grateful for support from the Bill & Melinda Gates Foundation and would like to thank the country teams and resident enumerators who are ultimately responsible for the success of PMA.

Figure 1a. Implants and injectables as a percent of the total modern contraceptive method mix, Burkina Faso

Figure 1b. Implants and injectables as a percent of the total modern contraceptive method mix, Kenya

Figure 2a. Percent of current implant users reporting unsuccessful discontinuation attempt

Figure 2b. Percent of all discontinuation attempts that were unsuccessful

Figure 3a. Reasons for implant discontinuation, Burkina Faso

Figure 3b. Reasons for implant discontinuation, Kenya

Figure 4. Location of implant discontinuation and discontinuation attempt in Burkina Faso and Kenya, 2017-2019

Figure 5a. Barriers to implant discontinuation, Burkina Faso

Figure 5b. Barriers to implant discontinuation, Kenya

For peer review only

References

1. Tumlinson K, Steiner MJ, Rademacher KH, Olawo A, Solomon M, Bratt J. The promise of affordable implants: is cost recovery possible in Kenya? Contraception. 2011 Jan;83(1):88–93.

2. Jacobstein R. Liftoff: the blossoming of contraceptive implant use in africa. Glob Health Sci Pract. 2018 Mar 21;6(1):17–39.

3. Winner B, Peipert JF, Zhao Q, Buckel C, Madden T, Allsworth JE, et al. Effectiveness of Long-Acting Reversible Contraception. Obstet Gynecol Surv. 2012 Sep;67(9):552–3.

4. Bahamondes L, Fernandes A, Monteiro I, Bahamondes MV. Long-acting reversible contraceptive (LARCs) methods. Best Pract Res Clin Obstet Gynaecol. 2020 Jul;66:28–40.

5. Kungu W, Khasakhala A, Agwanda A. Use of long-acting reversible contraception among adolescents and young women in Kenya. PLoS ONE. 2020 Nov 10;15(11):e0241506.

6. Kiemtoré S, Zoungrana Z, Zamané H, Kaboré CWPD, Ouédraogo A, Bonané B. Interventions to improve the use of long-acting reversible contraceptive methods at primary health centers in Burkina Faso. Int J Gynaecol Obstet. 2019 Dec;147(3):350–5.

7. Radovich E, Dennis ML, Barasa E, Cavallaro FL, Wong KL, Borghi J, et al. Who pays and how much? A cross-sectional study of out-of-pocket payment for modern contraception in Kenya. BMJ Open. 2019 Feb 20;9(2):e022414.

8. Ensuring human rights in the provision of contraceptive information and services: guidance and recommendations. Geneva: World Health Organization; 2014.

9. Christofield M, Lacoste M. Accessible Contraceptive Implant Removal Services: An Essential Element of Quality Service Delivery and Scale-Up. Glob Health Sci Pract. 2016 Sep 28;4(3):366–72.

10. Senderowicz L. "I was obligated to accept": A qualitative exploration of contraceptive coercion. Soc Sci Med. 2019 Oct;239:112531.

11. Britton LE, Williams CR, Onyango D, Wambua D, Tumlinson K. "When it comes to time of removal, nothing is straightforward": A qualitative study of experiences with barriers to removal of long-acting reversible contraception in Western Kenya. Contraception: X. 2021 Mar 22;3:100063.

12. Brunie A, Callahan RL, Mackenzie A, Kibira SPS, Wayack-Pambè M. Developing acceptable contraceptive methods: Mixed-method findings on preferred method

For peer review only

characteristics from Burkina Faso and Uganda. [version 2; peer review: 2 approved]. Gates Open Res. 2019 Apr 11;3:1205.

13. Zimmerman L, Olson H, PMA2020 Principal Investigators Group, Tsui A, Radloff S. PMA2020: Rapid Turn-Around Survey Data to Monitor Family Planning Service and Practice in Ten Countries. Stud Fam Plann. 2017 Aug 28;48(3):293–303.

14. World Health Organization Department of Reproductive Health and Research (WHO/RHR) and Johns Hopkins Bloomberg School of Public Health/Center for Communication Programs (CCP), Knowledge for Health Project. Family Planning: A Global Handbook for Providers (2018 Update). Updated 3rd. Baltimore and Geneva: CCP and WHO; 2021.

15. Festin MPR, Kiarie J, Solo J, Spieler J, Malarcher S, Van Look PFA, et al. Moving towards the goals of FP2020 - classifying contraceptives. Contraception. 2016 Oct;94(4):289–94.

16. Yirgu R, Wood SN, Karp C, Tsui A, Moreau C. "You better use the safer one… leave this one": the role of health providers in women's pursuit of their preferred family planning methods. BMC Womens Health. 2020 Aug 12;20(1):170.

17. Senderowicz L, Kolenda A. "She told me no, that you cannot change": Understanding provider refusal to remove contraceptive implants. SSM - Qualitative Research in Health. 2022 Dec;2:100154.

18. Senderowicz L, Karp C, Bullington BW, Tumlinson K, Zimmerman L, OlaOlorun FM, et al. Facility readiness to remove subdermal contraceptive implants in 6 sub-Saharan African countries. AJOG Glob Rep. 2022 Nov 5;2(4):100132.

19. Tumlinson K, Britton LE, Williams CR, Wambua DM, Otieno Onyango D. Informal payments for family planning: prevalence and perspectives of women, providers, and health sector key informants in western Kenya. Sexual and Reproductive Health Matters. 2021 Dec;29(1):1–17.

20. Brunie A, Stankevitz K, Nwala AA, Nqumayo M, Chen M, Danna K, et al. Expanding long-acting contraceptive options: a prospective cohort study of the hormonal intrauterine device, copper intrauterine device, and implants in Nigeria and Zambia. Lancet Glob Health. 2021 Oct;9(10):e1431–41.

21. Callahan R, Lebetkin E, Brennan C, Kuffour E, Boateng A, Tagoe S, et al. What Goes In Must Come Out: A Mixed-Method Study of Access to Contraceptive Implant Removal Services in Ghana. Glob Health Sci Pract. 2020 Jun 30;8(2):220–38.

For peer review only

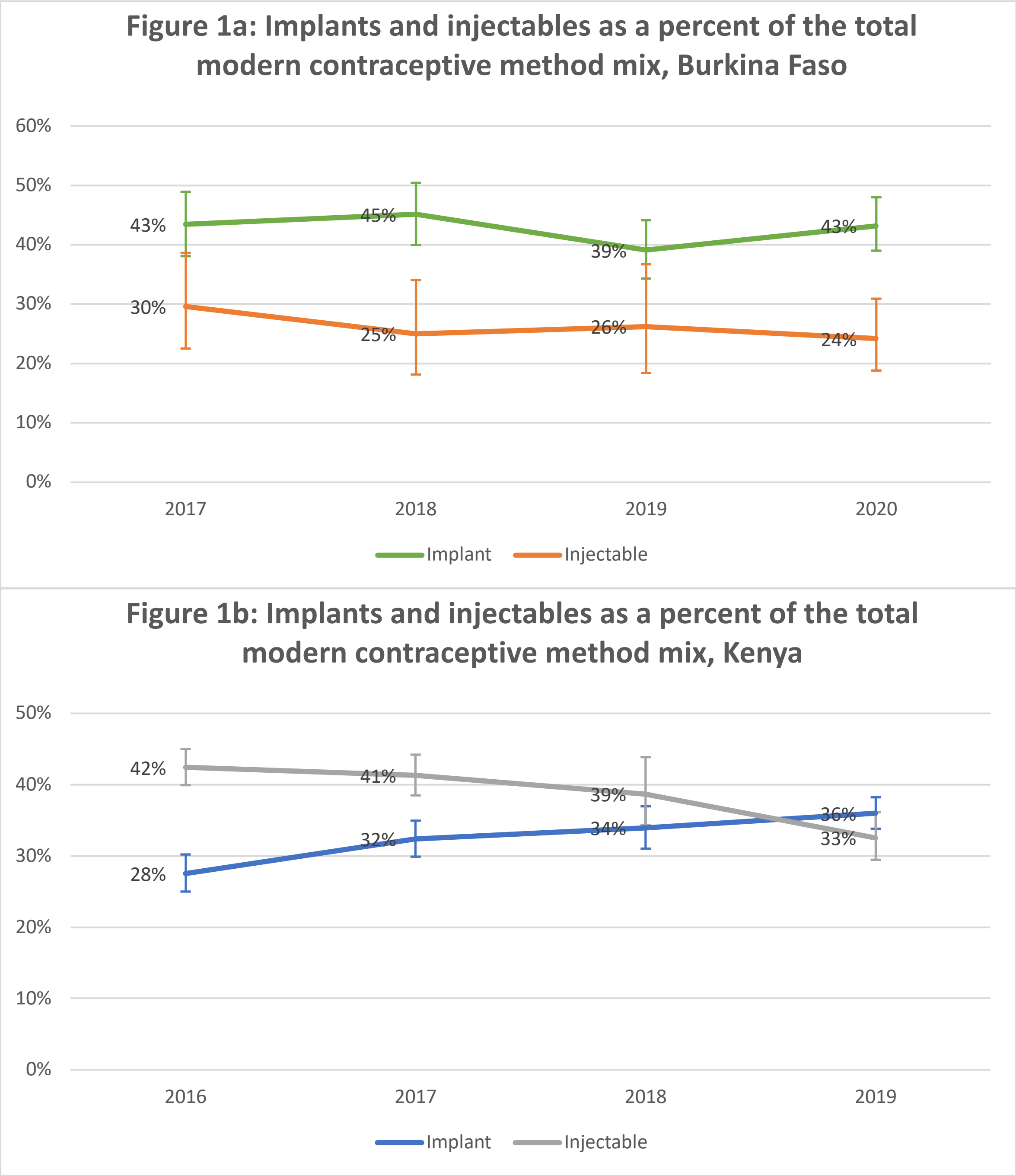

Figure 1a: Implants and injectables as a percent of the total modern contraceptive method mix, Burkina Faso

Figure 1b: Implants and injectables as a percent of the total modern contraceptive method mix, Kenya

Figure 2a: Percent of current implant users reporting unsuccessful discontinuation attempt*

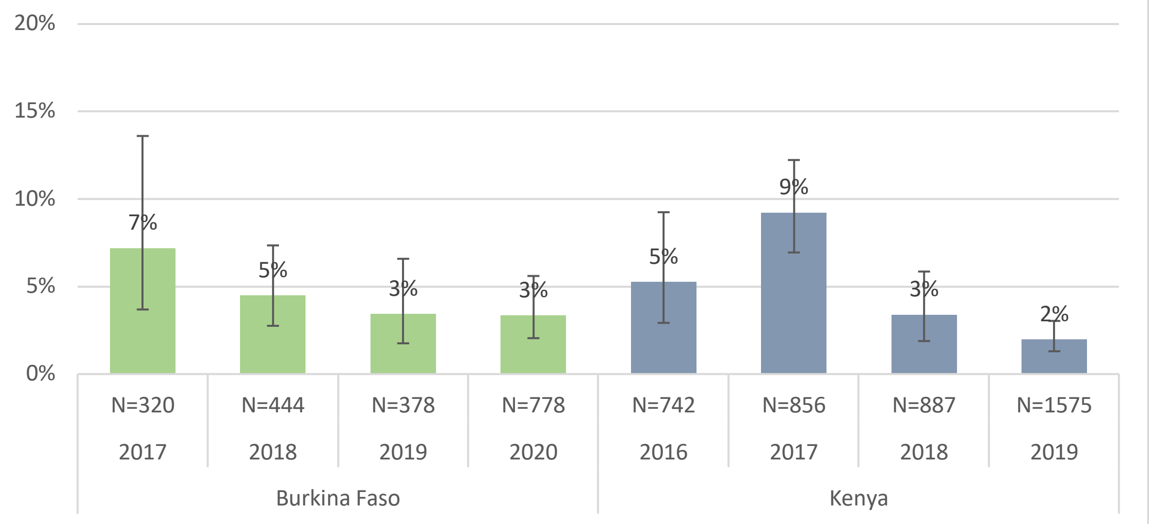

*Denominator is all current implant users.

Figure 2b: Percent of all discontinuation attempts that were unsuccessful*

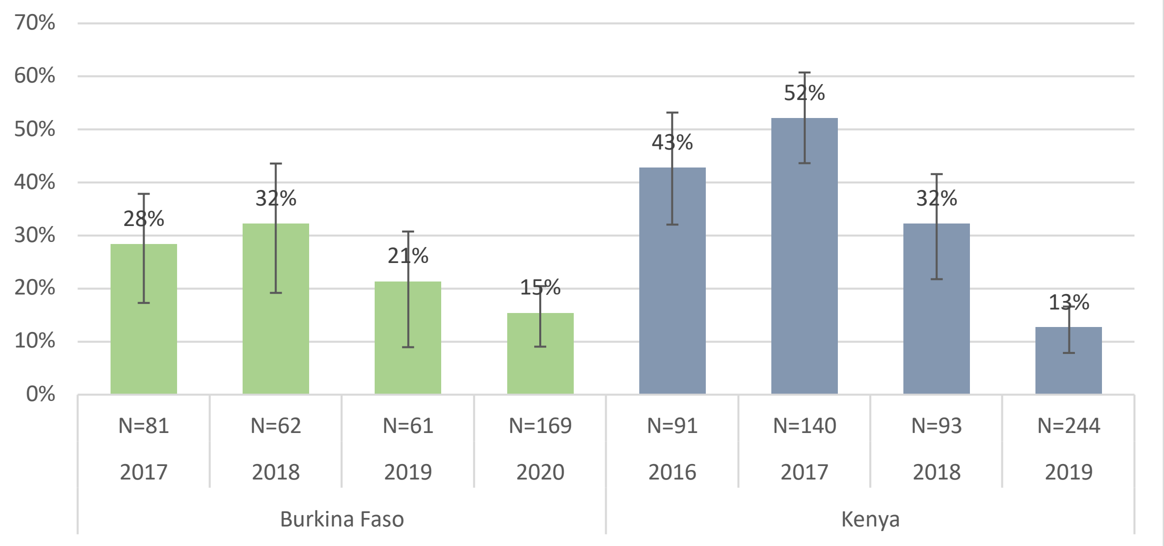

*Denominator is women with successful implant removal in the last 12 months and women with unsuccessful implant removal in the last 12 months. Due to limitations of the existing data, we are unable to estimate the proportion of successful removal among currently contracepting women.

Figure 3a: Reasons for implant discontinuation, Burkina Faso

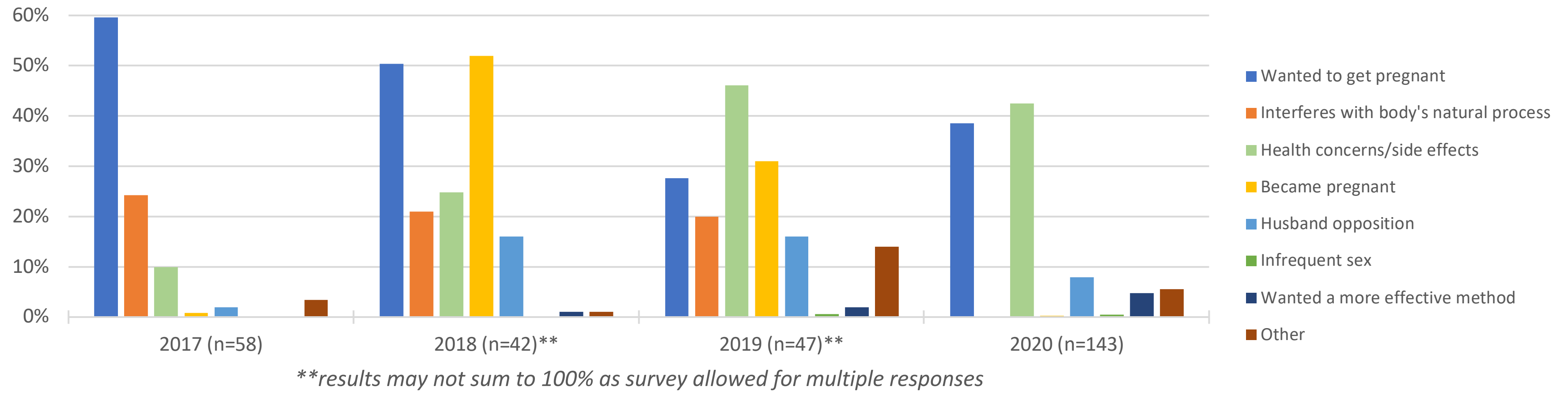

Figure 3b: Reasons for implant discontinuation, Kenya

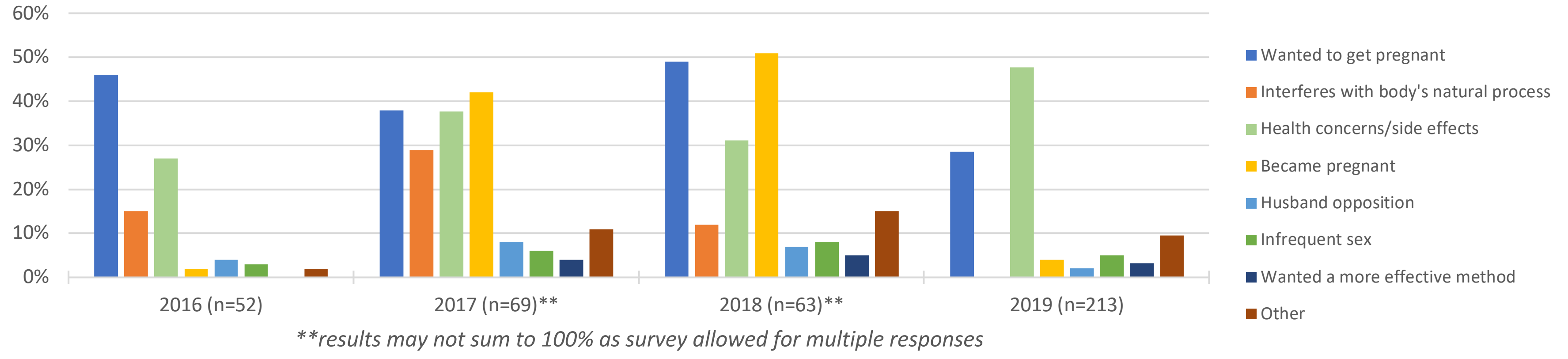

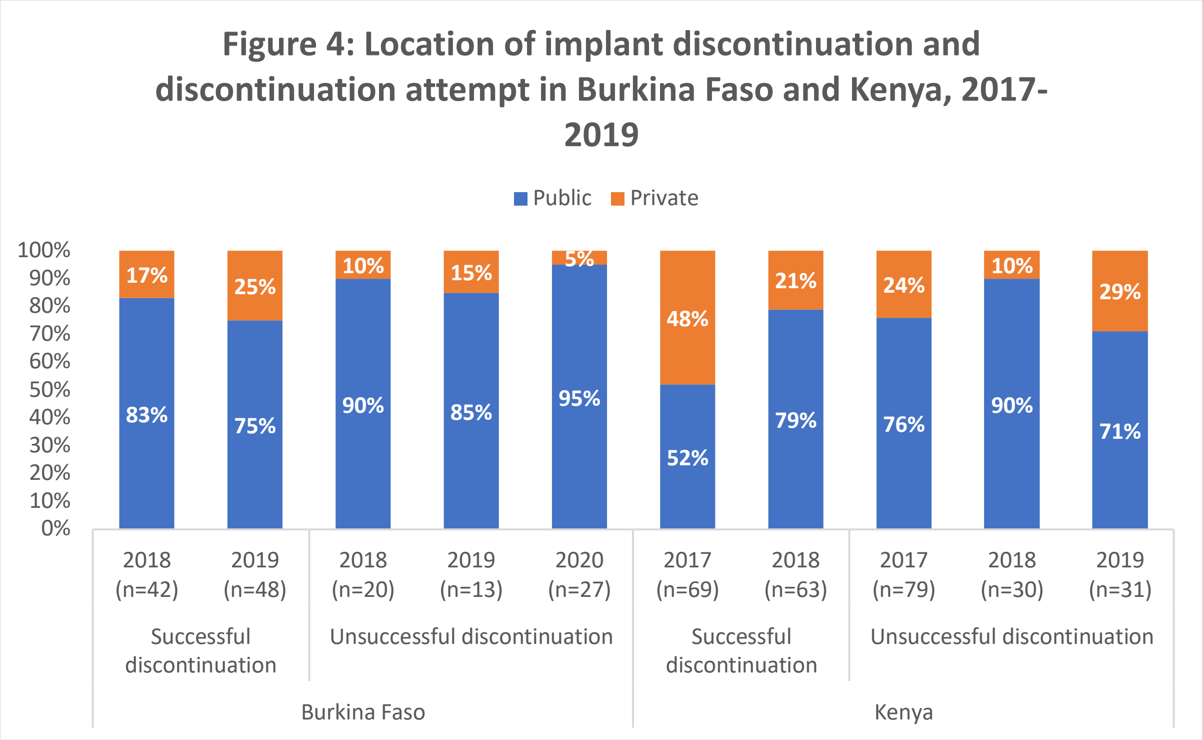
Figure 4: Location of implant discontinuation and discontinuation attempt in Burkina Faso and Kenya, 2017-2019
Public
Private
100%
90%
80%
70%
60%
50%
40%
30%
20%
10%
0%
17%
25%
10%
15%
5%
48%
21%
24%
10%
29%
83%
75%
90%
85%
95%
52%
79%
76%
90%
71%
2018 (n=42)
2019 (n=48)
2018 (n=20)
2019 (n=13)
2020 (n=27)
2017 (n=69)
2018 (n=63)
2017 (n=79)
2018 (n=30)
2019 (n=31)
Successful discontinuation
Unsuccessful discontinuation
Successful discontinuation
Unsuccessful discontinuation
Burkina Faso
Kenya

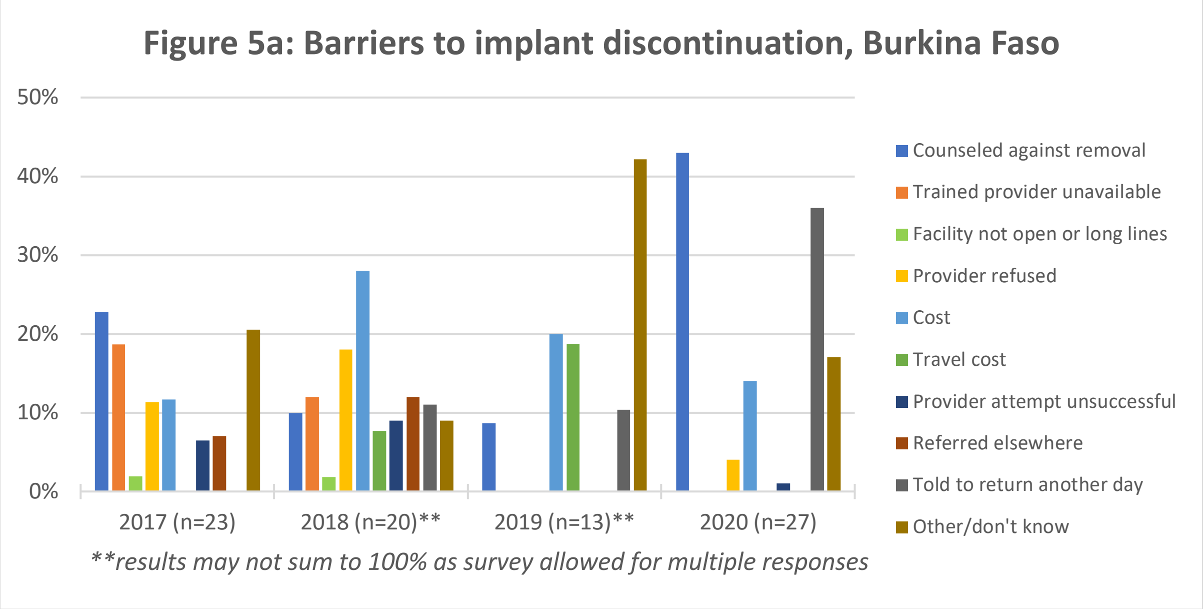
Figure 5a: Barriers to implant discontinuation, Burkina Faso
50%
40%
30%
20%
10%
0%
2017 (n=23)
2018 (n=20)**
2019 (n=13)**
2020 (n=27)
**results may not sum to 100% as survey allowed for multiple responses
Counseled against removal
Trained provider unavailable
Facility not open or long lines
Provider refused
Cost
Travel cost
Provider attempt unsuccessful
Referred elsewhere
Told to return another day
Other/don't know

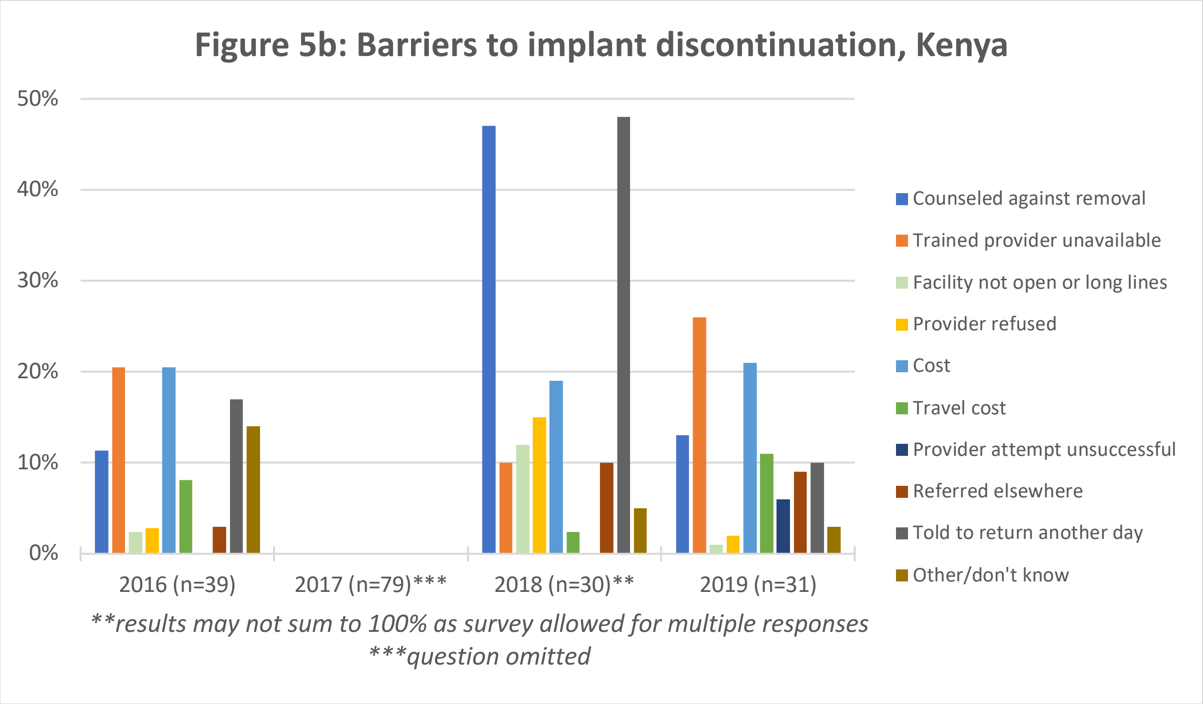
Figure 5b: Barriers to implant discontinuation, Kenya
50%
40%
30%
20%
10%
0%
2016 (n=39)
2017 (n=79)***
2018 (n=30)**
2019 (n=31)
**results may not sum to 100% as survey allowed for multiple responses
***question omitted
Counseled against removal
Trained provider unavailable
Facility not open or long lines
Provider refused
Cost
Travel cost
Provider attempt unsuccessful
Referred elsewhere
Told to return another day
Other/don't know

Supplemental Table 1. Characteristics of modern method users compared to implant users, in Burkina Faso and Kenya. 2019.

	Burkina Faso 2020			Kenya 2019		
	All Other Modern	Implant	*p-value	All Other Modern	Implant	*p-value
**N**	900	778		2463	1575	
**Total % of all women**	14%	12%		26%	17%	
**Age**			0.300			<0.001
15-24	31%	29%		25%	25%	
25-39	55%	50%		55%	61%	
40-49	14%	22%		20%	14%	
**Marital status**			<0.001			0.001
In union	71%	82%		69%	78%	
Sexually active unmarried	29%	18%		31%	22%	
**Parity**			<0.001			<0.001
0	17%	4%		11%	1%	
1 to 2	29%	30%		37%	42%	
3 to 4	27%	25%		31%	34%	
5 or more	27%	41%		21%	23%	
**Residence**			<0.001			<0.001
Urban	37%	22%		36%	27%	
Rural	63%	78%		64%	73%	
**Education**			<0.001			<0.001
No education	46%	64%		2%	4%	
Primary	19%	19%		49%	57%	
Secondary	30%	16%		37%	30%	
Tertiary	4%	1%		13%	9%	
**Wealth tertile**			<0.001			<0.001
Lowest	25%	34%		28%	38%	
Middle	28%	34%		37%	36%	
Highest	47%	32%		35%	26%	
**Received from public facility**			<0.001			<0.001
Yes	74%	91%		61%	88%	
No	26%	9%		39%	12%	
**Fertility preferences**			0.090			<0.001
No more children	18%	25%		44%	48%	
Children in the next 2 years	35%	31%		16%	13%	
Children more than 2 years from now	38%	34%		33%	34%	
Says she can't get pregnant	1%	1%		2%	1%	
Undecided	8%	9%		5%	3%	
Other	1%	0%		0%	0%	

**We use the chi-square test to assess whether the distribution of characteristics differed based on whether a woman reported implant versus other modern method use.*

For peer review only

Supplemental Table 2. Contraceptive prevalence and method mix over time, among women of reproductive age in Burkina Faso and Kenya. 2016-2020

	Burkina Faso				Kenya			
	2017	2018	2019	2020	2016	2017	2018	2019
**N**	3221	3512	3329	6590	5891	5864	5658	9469
**Current contraceptive use**								
Yes	23%	28%	29%	27%	45%	45%	46%	45%
No	77%	72%	71%	73%	55%	55%	54%	55%
**Current modern contraceptive use**								
Yes	22%	26%	27%	25%	44%	44%	45%	43%
No	78%	74%	73%	75%	56%	56%	55%	57%
**N**	735	982	960	1800	2659	2592	2566	4271
**Method mix***								
Sterilization	0%	0%	0%	0%	4%	4%	4%	4%
IUD	3%	4%	4%	4%	5%	2%	4%	4%
Implant	43%	45%	39%	43%	28%	32%	34%	36%
Injectable	30%	25%	26%	24%	42%	41%	39%	33%
Pills	11%	11%	10%	9%	8%	8%	7%	7%
Condoms	8%	8%	13%	12%	6%	5%	5%	7%
Standard Days Method	0%	1%	0%	0%	0%	1%	0%	0%
Rhythm	3%	5%	5%	6%	2%	2%	2%	3%
Withdrawal	0%	0%	0%	1%	1%	1%	1%	1%
Emergency contraception	0%	0%	1%	0%	2%	3%	3%	2%
Other modern method	0%	0%	0%	1%	1%	0%	0%	1%
Other	1%	0%	1%	0%	0%	0%	0%	2%

*Among women reporting use of more than one method, only the most effective method is included in the calculation of the method mix.

For peer review only

STROBE 2007 (v4) Statement—Checklist of items that should be included in reports of *cross-sectional studies*

Section/Topic	Item #	Recommendation	Reported on page #
**Title and abstract**	1	(*a*) Indicate the study's design with a commonly used term in the title or the abstract	2
		(*b*) Provide in the abstract an informative and balanced summary of what was done and what was found	2
**Introduction**			
Background/rationale	2	Explain the scientific background and rationale for the investigation being reported	3
Objectives	3	State specific objectives, including any prespecified hypotheses	3
**Methods**			
Study design	4	Present key elements of study design early in the paper	4
Setting	5	Describe the setting, locations, and relevant dates, including periods of recruitment, exposure, follow-up, and data collection	4
Participants	6	(*a*) Give the eligibility criteria, and the sources and methods of selection of participants	4
Variables	7	Clearly define all outcomes, exposures, predictors, potential confounders, and effect modifiers. Give diagnostic criteria, if applicable	4-5
Data sources/ measurement	8*	For each variable of interest, give sources of data and details of methods of assessment (measurement). Describe comparability of assessment methods if there is more than one group	4-5
Bias	9	Describe any efforts to address potential sources of bias	*4-5*
Study size	10	Explain how the study size was arrived at	6
Quantitative variables	11	Explain how quantitative variables were handled in the analyses. If applicable, describe which groupings were chosen and why	4-5
Statistical methods	12	(*a*) Describe all statistical methods, including those used to control for confounding	5
		(*b*) Describe any methods used to examine subgroups and interactions	NA
		(*c*) Explain how missing data were addressed	4-5
		(*d*) If applicable, describe analytical methods taking account of sampling strategy	NA
		(*e*) Describe any sensitivity analyses	NA
**Results**			

Participants	13*	(a) Report numbers of individuals at each stage of study—eg numbers potentially eligible, examined for eligibility, confirmed eligible, included in the study, completing follow-up, and analysed	6
		(b) Give reasons for non-participation at each stage	6
		(c) Consider use of a flow diagram	NA
Descriptive data	14*	(a) Give characteristics of study participants (eg demographic, clinical, social) and information on exposures and potential confounders	6
		(b) Indicate number of participants with missing data for each variable of interest	6
Outcome data	15*	Report numbers of outcome events or summary measures	6-7
Main results	16	(*a*) Give unadjusted estimates and, if applicable, confounder-adjusted estimates and their precision (eg, 95% confidence interval). Make clear which confounders were adjusted for and why they were included	6-7
		(*b*) Report category boundaries when continuous variables were categorized	4-5
		(*c*) If relevant, consider translating estimates of relative risk into absolute risk for a meaningful time period	NA
Other analyses	17	Report other analyses done—eg analyses of subgroups and interactions, and sensitivity analyses	NA
**Discussion**			
Key results	18	Summarise key results with reference to study objectives	7
Limitations	19	Discuss limitations of the study, taking into account sources of potential bias or imprecision. Discuss both direction and magnitude of any potential bias	8
Interpretation	20	Give a cautious overall interpretation of results considering objectives, limitations, multiplicity of analyses, results from similar studies, and other relevant evidence	7-8
Generalisability	21	Discuss the generalisability (external validity) of the study results	7-8
**Other information**			
Funding	22	Give the source of funding and the role of the funders for the present study and, if applicable, for the original study on which the present article is based	9

*Give information separately for cases and controls in case-control studies and, if applicable, for exposed and unexposed groups in cohort and cross-sectional studies.

Note: An Explanation and Elaboration article discusses each checklist item and gives methodological background and published examples of transparent reporting. The STROBE checklist is best used in conjunction with this article (freely available on the Web sites of PLoS Medicine at http://www.plosmedicine.org/, Annals of Internal Medicine at http://www.annals.org/, and Epidemiology at http://www.epidem.com/). Information on the STROBE Initiative is available at www.strobe-statement.org.

For peer review only
